# Comprehensive evaluation of methods for small extracellular vesicles separation from human plasma, urine and cell culture medium

**DOI:** 10.1002/jev2.12044

**Published:** 2021-01-15

**Authors:** Liang Dong, Richard C. Zieren, Kengo Horie, Chi‐Ju Kim, Emily Mallick, Yuezhou Jing, Mingxiao Feng, Morgan D. Kuczler, Jordan Green, Sarah R. Amend, Kenneth W. Witwer, Theo M. de Reijke, Yoon‐Kyoung Cho, Kenneth J. Pienta, Wei Xue

**Affiliations:** ^1^ The Brady Urological Institute Johns Hopkins University School of Medicine Baltimore Maryland USA; ^2^ Department of Urology, Renji Hospital Shanghai Jiao Tong University School of Medicine Shanghai China; ^3^ Department of Urology, Amsterdam UMC University of Amsterdam Amsterdam the Netherlands; ^4^ Department of Urology Gifu University Graduate School of Medicine Gifu Japan; ^5^ Department of Biomedical Engineering, School of Life Sciences Ulsan National Institute of Science and Technology (UNIST) Ulsan Republic of Korea; ^6^ Department of Molecular and Comparative Pathobiology Johns Hopkins University School of Medicine Baltimore Maryland USA; ^7^ Department of Biomedical Engineering, Institute for NanoBioTechnology, and Translational Tissue Engineering Center Johns Hopkins University School of Medicine Baltimore Maryland USA; ^8^ Department of Neurology Johns Hopkins University School of Medicine Baltimore Maryland USA; ^9^ Center for Soft and Living Matter Institute for Basic Science (IBS) Ulsan Republic of Korea

**Keywords:** EV yield, methods comparison, sample purity, single particle phenotyping, small extracellular vesicle separation

## Abstract

One of the challenges that restricts the evolving extracellular vesicle (EV) research field is the lack of a consensus method for EV separation. This may also explain the diversity of the experimental results, as co‐separated soluble proteins and lipoproteins may impede the interpretation of experimental findings. In this study, we comprehensively evaluated the EV yields and sample purities of three most popular EV separation methods, ultracentrifugation, precipitation and size exclusion chromatography combined with ultrafiltration, along with a microfluidic tangential flow filtration device, Exodisc, in three commonly used biological samples, cell culture medium, human urine and plasma. Single EV phenotyping and density‐gradient ultracentrifugation were used to understand the proportion of true EVs in particle separations. Our findings suggest Exodisc has the best EV yield though it may co‐separate contaminants when the non‐EV particle levels are high in input materials. We found no 100% pure EV preparations due to the overlap of their size and density with many non‐EV particles in biofluids. Precipitation has the lowest sample purity, regardless of sample type. The purities of the other techniques may vary in different sample types and are largely dependent on their working principles and the intrinsic composition of the input sample. Researchers should choose the proper separation method according to the sample type, downstream analysis and their working scenarios.

## 1 INTRODUCTION

Extracellular vesicles (EVs) are lipid bilayer encapsulated particles that can be released by all living cells into the extracellular space (Kalluri & LeBleu, 2020). EVs carry a variety of molecular cargos, including proteins, metabolites, and nucleic acids, and are considered to play an important role in cell‐to‐cell communication by transfer of their cargo to target cells locally as a means of paracrine signalling or by travelling to a distant body site (Becker et al., 2016, Valadi et al., 2007, Yokoi et al., 2019). EVs have been demonstrated to participate in a wide range of physiological and pathological processes, including immunomodulation, embryo implantation, pathogenic injury, and cancer (Cruz, Romero, Iglesia, & Lopes, 2018, Dong et al., 2019, Malloci et al., 2019). In the last decade there has been a dramatic increase in the number of publications on EVs, covering the field of basic biology, EVs as therapeutics, and the utility of EVs as biomarkers in liquid biopsies (Roy, Hochberg, & Jones, 2018).

Though the emerging work of EVs has generated much excitement, one of the biggest challenges restricting EV research is that despite the compelling findings of EV studies, the reproducibility and the concordance among studies is suboptimal (Yang et al., 2020). One reason for this variability is the diversity of EV separation methods used in different studies. EV separation is the foundation of EV work, determining what material will be further analyzed. Separation methods are based on different principles that may provide EV products containing distinct EV subpopulations with different levels of contaminants (Buschmann et al., 2018, Royo et al., 2016). The most commonly used EV separation methods are ultracentrifugation (UC), size exclusion chromatography (SEC) and polymer‐based precipitation (Gardiner et al., 2016). Many novel technologies have also emerged in recent years, most notably microfluidic‐based devices.

EV yield and purity are the two most essential considerations for an EV separation technique. The EV yield indicates how many EVs can be separated from a certain input material, regardless of contamination levels. The purity indicates the percentage of “true EVs” in the product relative to particle contamination. Ideally, the best separation method should have both high yield and high purity. Since the composition of different biofluids varies, it could be possible that the yield and purity of EV separation methods were different across sample types.

EVs separated from biofluids can be a highly heterogeneous population. Their heterogeneity can be reflective of their size, molecular cargo, functional impact and cellular origin (Kalluri & LeBleu, 2020). Conventional EV cargo analyses take a large pool of separated EVs as a whole, but it has been shown that not all EVs within a single sample contain the same abundance of a given cargo (Chevillet et al., 2014, Kowal et al., 2016, Willms, Cabañas, Mäger, Wood, & Vader, 2018). In recent years, with the help of a growing number of novel technologies allowing single particle analysis, for example, nano‐flow cytometry (nFCM), EV subpopulation analysis has been improved (Lian, He, Chen, & Yan, 2019, Welsh et al., 2020). However, the value of these technologies in evaluating EV separation methods needs to be explored.

In this study, we evaluated three EV separation methods, UC, precipitation and SEC combined with ultrafiltration (SEC+UF), along with a novel microfluidic tangential flow filtration device (mTFF), in three commonly used biological samples, cell culture medium (CCM), human urine, and human plasma (Gardiner et al., 2016). We demonstrated the performance of these EV separation methods for each of these sample types, specifically focusing on EV yield and purity.

## 2 MATERIALS AND METHODS

### 2.1 Preparation of conditioned cell culture medium

The human prostate cancer cell line PC3 was purchased from the American Type Culture Collection (ATCC, Manassas, VA, USA). PC3 cells were maintained in RPMI 1640 (Thermo Fisher Scientific, Waltham, MA, USA) containing 10% fetal bovine serum (FBS) (VWR, Radnor, PA, USA) and 5 U/ml Penicillin Streptomycin (Thermo Fisher Scientific). For EV separation, cells were grown in medium containing 10% exosome‐depleted FBS (Thermo Fisher Scientific) until they reached a confluency of ∼90% (after approximately 48 h). The cell number at CCM harvest was around 7.5×10^6^/T150 flask. Each flask was used to condition 20 ml of culture media. Ten to fifteen T150 flasks were used for each experiment (the input volume of CCM for each method was different and details can be found in each separation method section). First, the fresh CCM was immediately centrifuged at 1000 × *g* for 10 min to eliminate cells and large debris. Second, the supernatant was centrifuged at 10,000 × *g* for 20 min at 4°C to remove small debris, apoptotic bodies and other large EVs. Third, the supernatant was filtered through a 0.45 μm hydrophilic PVDF membrane syringe filter (Thermo Fisher Scientific). The pre‐processed CCM was either used fresh for EV separation or stored at −80°C until use, limiting freeze‐thaw cycles to a maximum of one.

### 2.2 Preparation of human plasma

For EV separation from plasma, whole blood from fasted healthy donors was collected in an ethylenediaminetetraacetic acid (EDTA) coated tube (BD Biosciences, San Jose, CA, USA.). Separation of plasma from other components was achieved by centrifugation at 1000 × *g* for 10 min, after which the plasma was transferred to a new tube. The plasma was further pre‐cleaned by two centrifugations at 2500 × *g* for 15 min to remove cells, large debris, and thrombocytes with supernatant collected after each spin. The supernatant was then centrifuged at 10,000 × *g* at 4°C for 20 min to remove apoptotic bodies, other large EVs, and some non‐EVs components. For further reduction of lipoproteins, likely the most abundant source of plasma proteins, the supernatant was filtered using a 0.45 μm hydrophilic PVDF membrane syringe filter (Thermo Fisher Scientific). The plasma was either used freshly for EV separation or stored at −80°C for later use, limiting freeze‐thaw cycles to a maximum of one.

### 2.3 Preparation of human urine

Non‐first void urine samples were obtained from healthy donors. Pre‐processing steps were the same as previously described for CCM. Urine was pre‐cleaned by centrifugation at 1000 × *g* for 10 min. Next, the supernatant was centrifuged at 10,000 × *g* for 20 min at 4°C. Lastly, the supernatant was filtered through a 0.45 μm hydrophilic PVDF membrane syringe filter (Thermo Fisher Scientific) and stored at −80°C until use, limiting freeze‐thaw cycles to a maximum of one.

### 2.4 Ultracentrifugation

Technical protocols of 4 EV separation methods compared in this study were schematically diagrammed as shown in Figure S1. Fresh or thawed CCM, plasma, or urine were centrifuged at 120,000 × *g* for 2 h at 4°C. After the first UC spin, we used PBS to resuspend the EV pellet, followed by a second UC spin in the same tube at 120,000 × *g* for 2 h at 4°C. For EV separation from CCM and urine (100–150 ml), a Beckman Coulter Type 70 Ti fixed angle rotor was used (adjusted *k*‐factor 131, maximal acceleration, maximal deceleration). For EV separation from plasma (4 ml), the Beckman Coulter Type 70.1 Ti fixed angle rotor was used (adjusted *k*‐factor 102, maximal acceleration, maximal deceleration). After the removal of supernatant, the EV pellets were resuspended and collected in 100 μl PBS.

**FIGURE 1:**
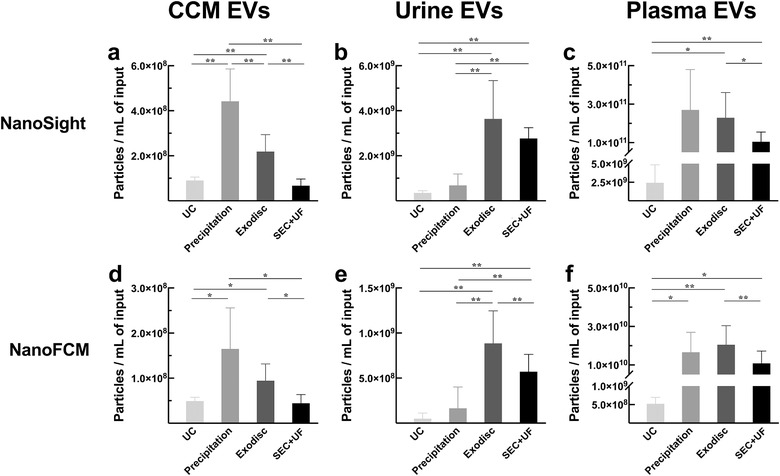
Concentrations of particles separated by different methods from CCM, urine and plasma, measured by NTA (a)‐(c) and nFCM (d)‐(f). The particle concentrations have been corrected for sample input volumes. (a) and (d) Particle concentrations of CCM EV preparations; (b) and (e) Particle concentrations of urine EV preparations; (c) and (f) Particle concentrations of plasma EV preparations. The error bars represented the standard deviation of five repetitive experiments. *P < 0.05, **P < 0.01, one‐way ANOVA analysis with Tukey multiple comparison test as well as a variance‐covariance model

### 2.5 Polymer‐based precipitation

Total Exosome Isolation Reagent appropriate for the sample type was used to separate EVs according to the protocols supplied by the manufacturer (Invitrogen, Carlsbad, CA, USA). Briefly, pre‐processed CCM (30 ml) was mixed with Total Exosome Isolation reagent (ratio: 1:0.5) and incubated at 4°C overnight. For urine samples (30 ml), the mixing ratio with Total Exosome Isolation reagent was 1:1. The mixture was then incubated for 1 h at room temperature (RT). After incubation, CCM or urine samples were centrifuged at 10,000 × *g* for 1 h at 4°C. The supernatant was carefully discarded, and the pellet was resuspended in 100 μl PBS. Pre‐processed plasma (0.5 ml) was mixed with 0.5 volume of PBS and 0.05 volume of proteinase K and incubated for 10 min at 37°C. Total Exosome Isolation reagent was then added to the proteinase K‐treated sample. After 30‐min incubation at 4°C, the mixture was centrifuged at 10,000 × *g* for 5 min at 4°C. The resulting pellet was resuspended in a total volume of 100 μl of PBS.

### 2.6 Exodisc

Exodisc is a centrifugal microfluidic device that can separate EVs by TFF with nano‐sized filters (Woo et al., 2017). The commercial versions of Exodisc (LabSpinner™, Ulsan, South Korea) were used for CCM and urine. Exodisc‐P for plasma was fabricated in accordance with the previously published work (Sunkara et al., 2019). Each Exodisc has 6 nano‐sized filters (anodic aluminum oxide membrane) with a diameter of 13 mm. The pore size of the membranes used for Exodisc for CCM and Urine was 20 nm, while it was 100 nm for Exodisc‐P (Sunkara et al., 2019). EV separation on Exodisc were done according to the instructions as shown in previous work (Sunkara et al., 2019, Woo et al., 2017). Briefly, biofluid samples (30 ml for CCM, 20 ml for urine and 0.5 ml for plasma) were applied to an Exodisc and processed using the bench‐top operating machine (OPR‐1000, LabSpinner™, South Korea). CCM and urine samples were centrifuged at 500 × *g* and plasma at 66 × *g*. Purified EVs were retrieved through the elution hole using 100 μl of PBS.

### 2.7 Size exclusion chromatography with ultrafiltration

For CCM and urine (40–60 ml), 10K molecular weight cut off (MWCO) Centricon^®^ Plus‐70 Centrifugal Filters (MilliporeSigma, Burlington, MA, USA) were used to concentrate the initial volume to 0.5 ml according to the manufacturer's instructions. qEV original 70 nm columns (IZON Science, Cambridge, MA) were used for EV separation. Briefly, the columns were brought to RT for 30 min and washed with PBS. Up to 0.5 ml of sample was loaded to the column. Fourteen sequential fractions of 0.5 ml were eluted by adding PBS. We identified EV enriched fractions (EVEF) as fractions 7 to 10 (Figure S2). EVEF were pooled and further concentrated using 10K MWCO Amicon^®^ Ultra‐2 Centrifugal Filters (MilliporeSigma) to a final volume of 100 μl.

**FIGURE 2:**
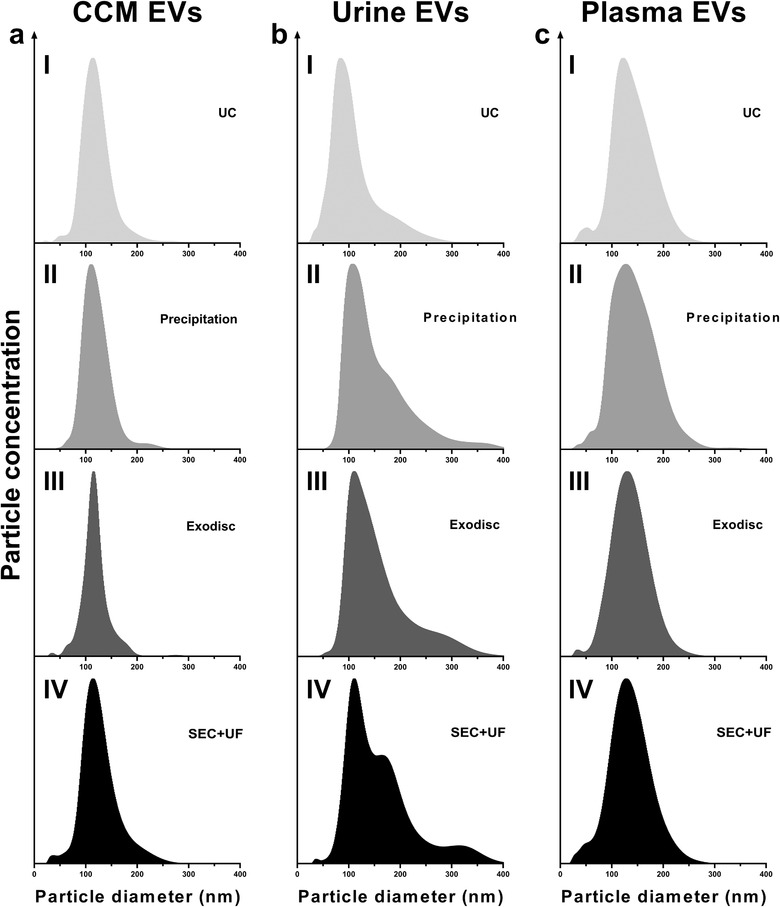
Particle size distributions of EV preparations separated by different methods from CCM, urine and plasma, measured by NTA. The concentration of particles in each bin of size was recorded. The bin width was 1.0 nm. In order to make the size distribution histogram visually comparable, the Y axis was adjusted to make the concentration of particles with modal size (the peak of the curve) as 95% of maximum scale in each figure. (a) Particle size distributions of CCM EV preparations; (b) Particle size distributions of urine EV preparations; (c) Particle size distributions of plasma EV preparations

### 2.8 Density gradient ultracentrifugation

After separation by various methods, EV pellets were resuspended in 2 ml of PBS and then loaded at the bottom of an ultra‐clear tube (Beckman Coulter). 60%, 50%, 40%, 30%, 25%, 15%, 10% and 5% w/v iodixanol solutions were prepared by diluting a stock solution (60% w/v) of iodixanol (OptiPrep™, Sigma # 1556) with a buffer solution (0.25 M sucrose//0.9 M NaCl/120 mM HEPES, pH 7.4). A discontinuous iodixanol gradient was generated by successively layering 4.5 ml of each iodixanol solution on top of the EV suspension. The tubes were then centrifuged at 100,000 × *g* for 4 h at 4°C in a SW32.1 Ti rotor (adjusted *k*‐factor 299.6, minimal acceleration, no deceleration). Eight fractions were then collected starting with the top fraction. The density of each fraction was assessed by measuring weight and volume. All fractions were diluted and washed with PBS and centrifuged at 120,000 × *g* for 2 h at 4°C in a 70 Ti rotor (adjusted *k*‐factor 113.7, maximal acceleration, maximal deceleration). The resulting pellets for each fraction were resuspended in 50 μL of PBS.

### 2.9 Transmission electron microscopy

Separated EVs were assessed by transmission electron microscopy (TEM) as described previously (Zieren et al., 2020). First, 10 μl of each sample was adsorbed to an ultra‐thin carbon coated 400 mesh copper grid that was glow discharged (EMS GloQube™) by floatation for 2 min. Then grids were quickly blotted on filter paper and rinsed three times in tris‐buffered saline (TBS) for 1 min. The grids were negatively stained in two consecutive drops of 1% uranyl acetate with methylcellulose (filtered twice through 0.22 μm filter). The excessive stain was quickly blotted and aspirated. When completely dried in darkness, the grids were visualized using a Philips CM‐120 TEM operating at 80 kV with an AMCT XR80 CCD sensor.

### 2.10 Nanoparticle tracking analysis

Nanoparticle tracking analysis (NTA) (NanoSight NS300, Malvern Pananalytical) was used to measure the particle counts and size distributions of EV preparations separated from different sample types using different methods, as described previously (Zieren et al., 2020). Briefly, 10 μl of each EV sample was diluted with PBS according to the detection range (20–100 particles/frame). Nanosight software (NTA 3.4 Build 3.4.003) recorded three 60‐second videos with settings as follows: syringe flow rate 50; screen gain 2; and camera level 9. The same software was used to analyse the videos with settings: screen gain 10 and detection threshold 4. The particle concentrations were corrected for the input sample volume, volume of EV resuspension, and dilution necessary for NTA‐reading.

### 2.11 Nano flow cytometer

EV samples were analyzed by the nFCM (NanoFCM, Xiamen, China) for particle concentration, size distribution and surface protein maker phenotyping according to reported protocols (Tian et al., 2018, Zhu et al., 2014). Briefly, two single photon counting avalanche photodiodes (APDs) were used for the simultaneous detection of side scatter (SSC) and fluorescence of individual particles. The instrument was calibrated for particle concentration using 200 nm PE and AF488 fluorophore conjugated polystyrene beads and for size distribution using Silica Nanosphere Cocktail (NanoFCM Inc., S16M‐Exo). Any particles that passed by the detector during a 1‐min interval were recorded in each test. All samples were diluted to attain a particle count within the optimal range of 2000–12,000/min. Using the calibration curve, the flow rate and side scattering intensity were converted into corresponding vesicle concentration and size on the NanoFCM software (NanoFCM Profession V1.0). It was not the purpose or focus of this study to do a head‐to‐head comparison between NTA and nFCM. Here they were taken as two independent particle characterization methods based on different principles to give parallel evaluation of EV products.

For EV surface protein marker phenotyping, the following antibodies were purchased from BD Biosciences: PE‐conjugated mouse anti‐human CD9 antibody (clone M‐L13), PE‐conjugated mouse anti‐human CD63 antibody (clone H5C6), PE‐conjugated mouse anti‐human CD81 antibody (clone JS‐81) and PE‐conjugated mouse IgG1, κ (clone MOCP‐21). An aliquot of EV samples separated by different methods was suspended in 20 μL of PBS with a particle concentration of 1×10^9^ particles/ml, respectively. Of each EV preparation, 20 μL was mixed with 20 μL PE‐conjugated antibody and incubated for 60 min at 37°C. After incubation, the mixture was washed with PBS and centrifuged at 120,000 × *g* for 2 h at 4°C. The pellet was then resuspended in 50 μL of PBS for phenotype analysis.

### 2.12 Murine virus produced in murine cells

Murine viral particles expressing superfolder green fluorescent protein on the outer surface of the viral envelope (MVP‐GFP) were purchased from ViroFlow Technologies Inc. (Ottawa, Cananda). The ecotropic murine retrovirus is produced in mouse fibroblasts. They can be detected on nFCM by both SSC and FITC channel, thus, was used as reference particles for the spiking experiment.

### 2.13 BCA assay

EV samples were lysed using 5×RIPA buffer (Cell Biolabs) with added HALT™ Proteinase and Phosphatase Inhibitor Cocktail (Thermo Fisher Scientific). Protein concentrations were determined using either the Pierce™ BCA Protein Assay or the Micro BCA™ Protein Assay (Thermo Fisher Scientific). The assays were applied according to the manufacturer's protocols.

### 2.14 Western blotting

Assessment of EV protein markers and non‐EV protein markers was performed by Western blotting (WB). Within each sample type, equal protein amounts from EV products separated by different methods were run on 12% SDS Mini‐PROTEAN^®^ TGX Stain‐Free™ Protein Gel (Bio‐Rad Laboratories) after boiling for 5 min at 95°C. All blots included relevant antibody control samples. For EV markers, membrane and cytosolic fractions of MCF7 breast cancer cells served as high and low controls, respectively. The subcellular fractions were acquired using Mem‐PER™ Plus Membrane Protein Extraction Kit according to the manufacturer's instructions (Thermo Fisher Scientific). Recombinant human Apolipoprotein A1 (APOA1) was purchased from Abcam (#ab50239) and served as positive control for APOA1 staining. Healthy donor's urine was disposed of cells by centrifugation, lysed using 5×RIPA and used as positive control for Tamm‐Horsfall protein (THP) staining. Both MCF7 subcellular fractions were negative controls for THP.

Depending on the compatibility with each antibody, one out of two WB platforms was used. For the EV markers CD63, CD81, and Flotillin‐1 and non‐EV marker Calnexin, samples were run under non‐reducing and denaturing conditions. These proteins were transferred to nitrocellulose membranes (Trans‐Blot® Turbo™ Mini Nitrocellulose, Bio‐Rad Laboratories) and incubated in 1× casein blocking buffer (Sigma‐Aldrich) for 1 h at RT. The nitrocellulose membranes were incubated overnight in 4°C with the following antibodies: CD63 (1:250, mouse monoclonal TDS63, #10628D, Thermo Fisher Scientific); CD81 (1:100, mouse monoclonal 1.3.3.22, #sc‐7637, Santa Cruz Biotechnology); Flotillin‐1 (1:2,500, rabbit monoclonal EPR604, #ab133497, Abcam); and Calnexin (1:2,500, rabbit polyclonal, #ab22595, Abcam). The anti‐GAPDH (1:2,500, rabbit monoclonal 14C10, #2118, Cell Signaling Technology) was to show proper loading and running of the cytosolic MCF7 fraction. After incubation with primary antibodies, the membranes were washed with 0.1% Tween in tris‐buffered saline (TBST) three times for 10 min. Incubation with secondary antibodies was performed at RT for 1 h with IRDye® 680RD Goat anti‐Mouse IgG (1:20,000, #92668070, LI‐COR Biosciences) and IRDye® 800CW Goat anti‐Rabbit IgG (1:15,000, #92632211, LI‐COR Biosciences) followed by three additional 10‐min washes in TBST. The blots were imaged using the Odyssey® 9120 Infrared Imaging System (LI‐COR Biosciences).

For non‐EV markers THP and APOA1, protein lysates were run under reducing and denaturing conditions followed by transfer to PVDF membranes (Trans‐Blot® Turbo™ Mini PVDF, Bio‐Rad Laboratories) and blocking in 2% bovine serum albumin in TBST. Overnight incubation at 4°C of the membranes was performed with the following primary antibodies: THP (1:1,000, mouse monoclonal B‐2, #sc‐271022 Santa Cruz Biotechnology); and APOA1 (1:5,000, goat polyclonal, #ab7613, Abcam). After three TBST‐washes, the membranes were incubated with relevant HRP‐linked secondary antibodies for 1 h at RT; either goat anti‐rabbit IgG (1:3,000, #7074, Cell Signaling Technology), goat anti‐mouse IgG (1:3000, #7076, Cell Signaling Technology), or donkey anti‐goat IgG (1:15,000, ab205723, Abcam). The same anti‐GAPDH (1:2,500, rabbit monoclonal 14C10, #2118, Cell Signaling Technology) was to show proper loading and running of the cytosolic MCF7 fraction. After three more TBST‐washes, the membranes were incubated with SuperSignal™ West Dura Extended Duration Substrate (Thermo Fisher Scientific) for THP and APOA1 before imaging using the ChemiDoc™ XRS+ System (Bio‐Rad Laboratories).

### 2.15 RNA extraction and quantification

Total RNA from EV preparations was isolated using the miRNeasy micro kit (Qiagen, Hilden, Germany), according to the manufacturer´s instructions. RNA samples were eluted in 20 μL of RNAse‐free water and stored at −80°C. The isolated RNA was measured by capillary electrophoresis using an Agilent Bioanalyzer 2100 (Agilent Technologies, Santa Clara, CA, USA). RNA from each sample was denatured at 72°C for 2 min and loaded into RNA 6000 Pico total RNA kits (Agilent Technologies) to analyse RNA profile and concentration.

### 2.16 Statistical analysis

All experiments were done with three independent biological replicates and each biological replicate was tested for 4 EV separation methods at the same time. Data were shown as mean ± standard deviation (SD). Statistically significant differences of outcome measurements between 4 EV separation methods were assessed by one‐way ANOVA analysis with Tukey multiple comparison test as well as a variance‐covariance model that incorporates correlations of outcome measurements due to the same biological replicate used for 4 EV separation methods. *P* value of < 0.05 was considered significant. All statistical analyses were done in SAS (9.4) (SAS institute, Cary, NC, USA).

### 2.17 EV‐TRACK

We have submitted all relevant data of our experiments to the EV‐TRACK knowledgebase (EV‐TRACK ID: EV200092 and EV200093) (Van Deun et al., 2017).

## 3 RESULTS

### 3.1 Particle yields from different sample types

NTA and nFCM were used to analyse concentrations and size distributions of particles separated by different methods from CCM, urine and plasma. It should be noted that these counted particles were not necessarily all EVs. There were different types and amounts of contaminants co‐ separated by each method in different sample types. The particle concentrations have been corrected for the sample input volumes (Table S1). NTA and nFCM depicted similar trends among 4 methods in each sample type, but the concentrations measured by nFCM were consistently lower than those measured by NTA (Figure 1). In CCM, precipitation had the highest particle yield, followed by Exodisc. UC and SEC+UF had similar yields, about 4 times lower than that of precipitation. In urine, Exodisc had the highest particle yield, followed by SEC+UF. UC had the lowest yield which was 10–20 times lower than that of Exodisc. There was a dramatic difference in particle yields among methods in plasma. Precipitation and Exodisc had similarly high particle yields, which were about 100 times higher than that of UC.

### 3.2 Particle size distribution

**FIGURE 3:**
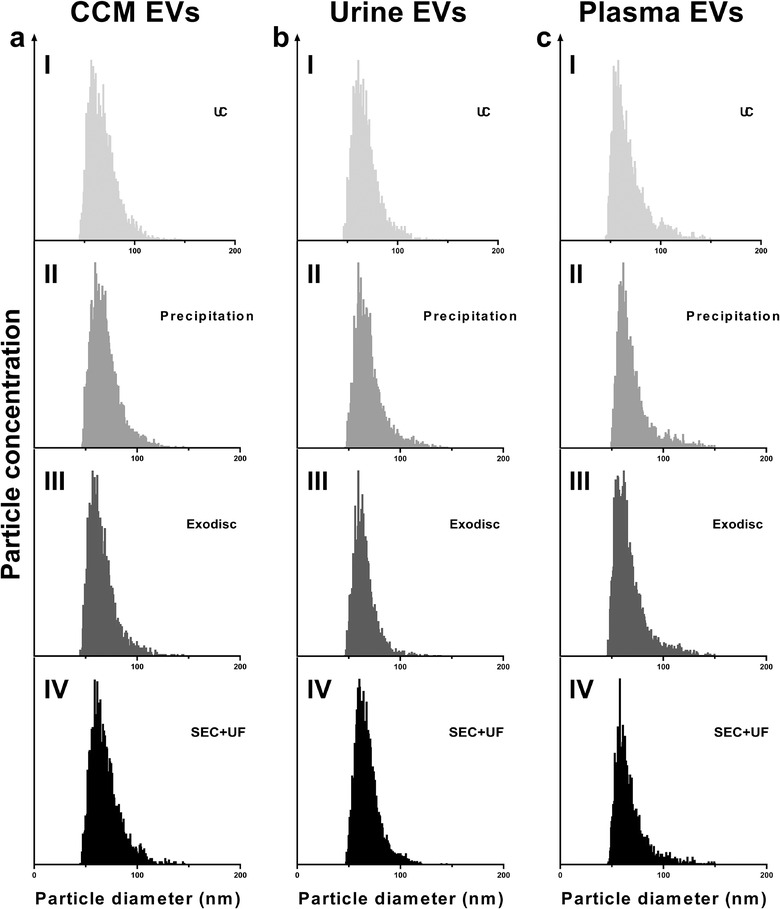
Particle size distributions of EV preparations separated by different methods from CCM, urine and plasma, measured by nFCM. The bin width was 0.5 nm. In order to make the size distribution histogram visually comparable, the Y axis was adjusted to make the concentration of particles with modal size (the peak of the curve) as 95% of maximum scale in each figure. (a) Particle size distributions of CCM EV preparations; (b) Particle size distributions of urine EV preparations; (c) Particle size distributions of plasma EV preparations

Particle size distributions were assessed by NTA and nFCM. The concentration of particles in each bin of size was recorded. The modal particle sizes measured by NTA were 113.5 ± 3.6 nm for UC, 110.5 ± 4.1 nm for precipitation, 115.5 ± 3.2 nm for Exodisc and 114.5 ± 3.8 nm for SEC+UF in CCM EV preparations (Figure 2a); 83.5 ± 4.2 nm for UC, 107.5 ± 2.6 nm for precipitation, 110.5 ± 3.1 nm for Exodisc and 110.5 ± 3.6 nm for SEC+UF in urine EV preparations (Figure 2b); 121.5 ± 3.2 nm for UC, 127.5 ± 3.2 nm for precipitation, 129.5 ± 3.5 nm for Exodisc and 128.5 ± 3.1 nm for SEC+UF in plasma EV preparations (Figure 2c). The particle size distributions were distinct among CCM, urine and plasma, regardless of the separation methods. There was no significant difference among methods in the same sample type. In contrast, all sample types had similar particle size distributions measured by nFCM (the modes were all around 60 nm) (Table S2, Figure 3a‐c). Comparing NTA with nFCM, the ranges of particle sizes were constantly wider in NTA vs. nFCM (10–400 nm vs. 40–150 nm).

### 3.3 EV yield and purity evaluated by spiking experiment

**FIGURE 4:**
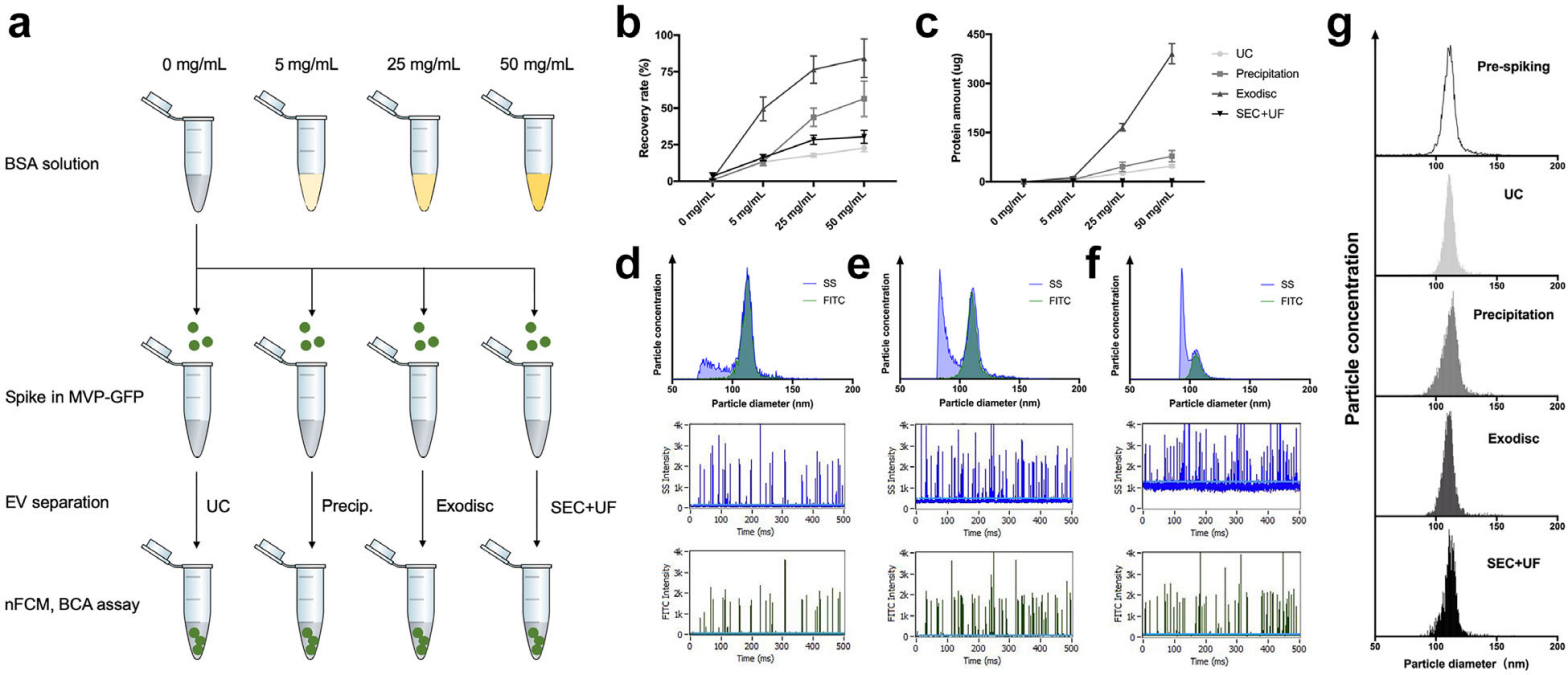
EV yield and purity evaluated by spiking experiment. (a) Schematic diagram of the experiment design. (b) The recovery rates of different separation methods in different contaminating protein concentration groups. The error bars represented the standard deviation of three repetitive experiments. (c) The co‐separated protein amounts in post‐separation samples by different separation methods in different contaminating protein concentration groups. The error bars represented the standard deviation of three repetitive experiments. (d) Particle size distribution histogram (top), SSC intensity plot (middle) and FITC intensity plot (bottom) of post‐separation sample by Exodisc when the protein concentration of pre‐separation sample is 5 mg/ ml. (e) Particle size distribution histogram (top), SSC intensity plot (middle) and FITC intensity plot (bottom) of post‐separation sample by Exodisc when the protein concentration of pre‐separation sample is 25 mg/ml. (f) Particle size distribution histogram (top), SSC intensity plot (middle) and FITC intensity plot (bottom) of post‐separation sample by Exodisc when the protein concentration of pre‐separation sample is 50 mg/ml. (g) Size distributions of recovered MVP‐GFP by different methods. In order to make the size distribution histogram visually comparable, the Y axis was adjusted to make the concentration of particles with modal size (the peak of the curve) as 95% of maximum scale in each figure

In order to compare the “true EV” yield among different methods, spiking experiments were performed using MVP‐GFP, whose fluorescence signal can be detected by nFCM. To mimic biofluid complexity, 2 × 10^7^ MVP‐GFP were spiked in 500 μL PBS supplemented with increasing concentrations of contaminating protein (bovine serum albumin), followed by EV separations using UC, precipitation, Exodisc and SEC+UF, respectively (Figure 4a). The number of spiked MVP‐GFP and recovered MVP‐GFP were all recorded using nFCM (FITC channel). The recovery rate was calculated by dividing the number of recovered MVP‐GFP by the number of spiked MVP‐GFP. The results demonstrated that very few MVP‐GFP can be recovered in pure PBS group in all four methods, probably because the chances for these particles to stick to surfaces are much higher in only PBS. The recovery rates increased with increasing concentrations of contaminating protein in pre‐separation samples in all four methods. When the concentration of contaminating protein in pre‐separation sample was 50 mg/ml, the recovery rate of Exodisc was 84.15 ± 13.22%, followed by precipitation (56.44 ± 12.12%), SEC+UF (30.43 ± 4.40%), and UC (22.72 ± 2.54%) (Figure 4b). On the other hand, we measured the protein concentrations for all post‐separation samples, which directly reflected the co‐separated contamination levels. The co‐separated protein levels were low in all four methods when the protein concentration of pre‐separation sample was 5 mg/ml (similar as CCM). When the protein concentration of pre‐separation sample was 50 mg/ml (similar as plasma), the co‐separated protein level got dramatically increased in Exodisc (Figure 4c). On particle size distribution histogram, when we overlapped SSC plot (any particles including co‐separated proteins and MVP‐GFP, blue) and FITC plot (only MVP‐GFP, green), an increasing number of small‐sized contaminants can be seen in post‐separation samples by Exodisc when the protein concentrations of pre‐separation samples increased (Figure 4d‐f). Since the sample background level also got higher in SSC intensity plots, a lot of small particles could be missed, which explained the sharp left edge of the SSC size distribution histogram (Figure 4e and f). Last but not least, the EV size distribution could be altered if a separation method only captures a subgroup of EVs with certain sizes, form aggregates, or add non‐EV particles to the product during separation. In this study, there was no difference between modal particle size before and after separation using any method (Figure 4g).

### 3.4 Purity of EV samples assessed by transmission electron microscopy

**FIGURE 5:**
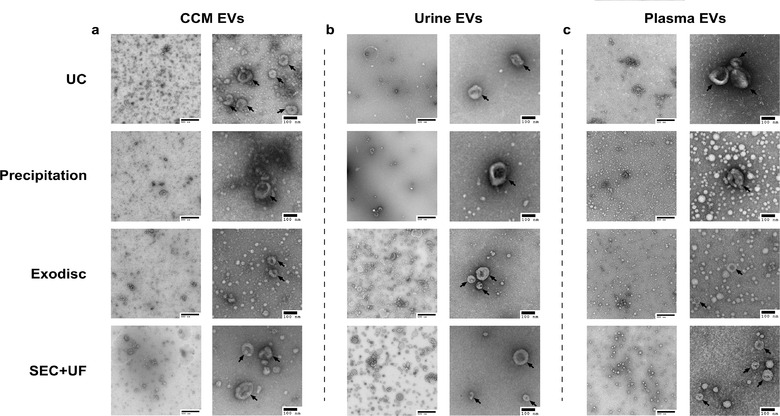
TEM images confirmed presence of negative‐stained EVs, seen as cup‐shaped vesicles. Representative image of (a) CCM EV preparations, (b) urine EV preparations, (c) plasma EV preparations separated by different methods. Scale bar represented 500 nm in figures with low magnifications on the left side and 100 nm in figures with high magnifications on the right side in each sample. Black arrows pointed out particles with a morphology that meets the criteria of EV

On TEM images of negatively stained EVs, we observed cup‐shaped particles in different sizes. The cup shape indicates an intact bilipid membranous vesicle, but dehydrated and, therefore, not perfectly spherical. In some of the images, non‐EV particles (i.e., lipids and protein aggregates) were also observed. In CCM, the majority of particles were cup‐shaped, surrounded by a few background non‐EV particles (Figure 5a). Urine samples generally had a very clear background (Figure 5b). EV samples prepared by precipitation had more non‐EV particles. In plasma, the majority of particles were non‐EV particles. Especially in precipitation and Exodisc samples, cup‐shaped EVs were rare (Figure 5c).

### 3.5 Purity of EV samples assessed by particle/protein ratio, RNA/protein ratio and Western blotting

**FIGURE 6:**
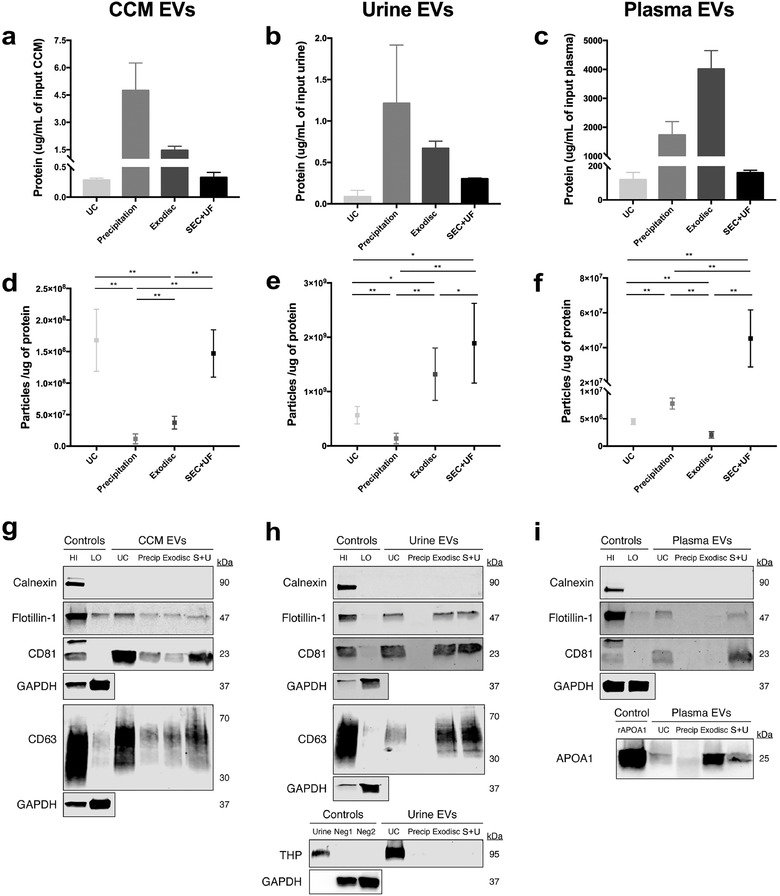
Purity of EV samples assessed by particle/protein ratio and Western blotting. (a)‐(c) The protein concentrations of EV preparations separated by different methods from CCM, urine and plasma. The protein concentrations have been corrected for initial sample input volume. (d)‐(f) The particle/protein ratios of EV preparations separated by different methods from CCM, urine and plasma. The particle numbers were measured by nFCM. The error bars represented the standard deviation of three repetitive experiments. *P < 0.05, **P < 0.01, one‐way ANOVA analysis with Tukey multiple comparison test as well as a variance‐covariance model. (g) Western blots of flotillin‐1, CD63, CD81, and calnexin for EV preparations separated from CCM by different methods. 9 μg of protein from each EV preparation was loaded. (h) Western blots of flotillin‐1, CD63, CD81, THP and calnexin for EV preparations separated from urine by different methods. 3.3 μg of protein from each EV preparation was loaded. (i) Western blots of flotillin‐1, CD81, APOA1 and calnexin for EV preparations separated from plasma by different methods. 25 μg of protein from each EV preparation was loaded. MCF7 membrane and cytosolic protein fractions served as positive and negative controls for flotillin‐1, CD63, CD81 and calnexin. Whole urine sample was used as positive control and MCF7 cellular fractions as negative control for THP. Recombinant human Apolipoprotein A1 served as positive control for APOA1.GAPDH staining was to show proper loading and running of the cytosolic MCF7 fraction

In order to evaluate the purity of EV products separated by different methods, the protein concentrations of EV samples corrected for initial sample input volume were measured (Table S3). The protein concentrations were higher overall in plasma EVs, compared to CCM and urine (Figure 6a‐c). The ultra‐high protein concentrations and the appearance of particles separated from plasma (Figure S3) indicated the likelihood for a high level of contamination. Since co‐separated free proteins have a big contribution to the total protein in EV preparations and not all the free proteins can be detected/counted by nFCM (i.e., smaller than the detectable range), the particle/protein ratio was calculated to demonstrate the purity of EV preparations (Table S3). In CCM, UC had the highest ratio, followed by SEC+UF. Exodisc and precipitation had relatively low ratios (Figure 6d). In general, urine samples had higher particle/protein ratios than other two sample types. SEC+UF had the highest ratio, followed by Exodisc and UC. The ratio of precipitation was the lowest (Figure 6e). Notably, the particle/protein ratios of plasma EVs were generally low, but the ratio for SEC+UF is significantly higher than the rest (Figure 6f). We also extracted total RNA from EV preparations and got the same finding by evaluating RNA/protein ratio. The only difference is that the RNA/protein ratio of UC in urine was the second highest among the four methods, while its particle/protein ratio was the second lowest (Figure S4). Notably, according to the protocol of precipitation for plasma EV separation, proteinase K was used in sample pre‐treatment, which may decrease the total protein amount. Thus, the ratios of precipitation in plasma might be overestimated.

Following protein quantification, sample purity was assessed using WB. Three EV markers (CD63, CD81 and Flotillin‐1) were used to identify the presence of EVs. Since the same amount of protein from each sample was loaded, higher expression of these EV markers indicated a higher concentration of “true EVs” in the sample. Several non‐EV markers were also tested, including Calnexin, a protein associated with the endoplasmic reticulum and could be released by cells suffering mechanical damage and should not be present in EVs. To assess contamination levels in urine EV separation, THP, also known as uromodulin, was tested. THP is the most abundant protein in urine and could form aggregates that co‐precipitate EVs. Likewise, to assess contamination in plasma EV separation, ApoA1, the major protein component for high density lipoprotein (HDL) in plasma, was probed. Calnexin was negative in all tested EV preparations. In CCM, UC had the highest expression levels of EV markers, followed by SEC+UF. Both precipitation and Exodisc had low expression levels (Figure 6g). In urine, both Exodisc and SEC+UF had high expression levels of EV markers, followed by UC. Precipitation was negative for these markers. UC was the only method that was positive for contamination marker THP, which may potentially explain why UC samples had a higher RNA/protein ratio, since THP can trap EVs and form aggregates, thus decreasing the particle counts, but may not affect the RNA amounts (Figure 6h). In plasma, only UC and SEC+UF were positive for EV markers and SEC+UF had higher expression levels. All of the samples were positive for ApoA1, among which Exodisc had the highest expression level (Figure 6i).

### 3.6 Tetraspanins‐positive particles assessed by single particle phenotyping

**FIGURE 7:**
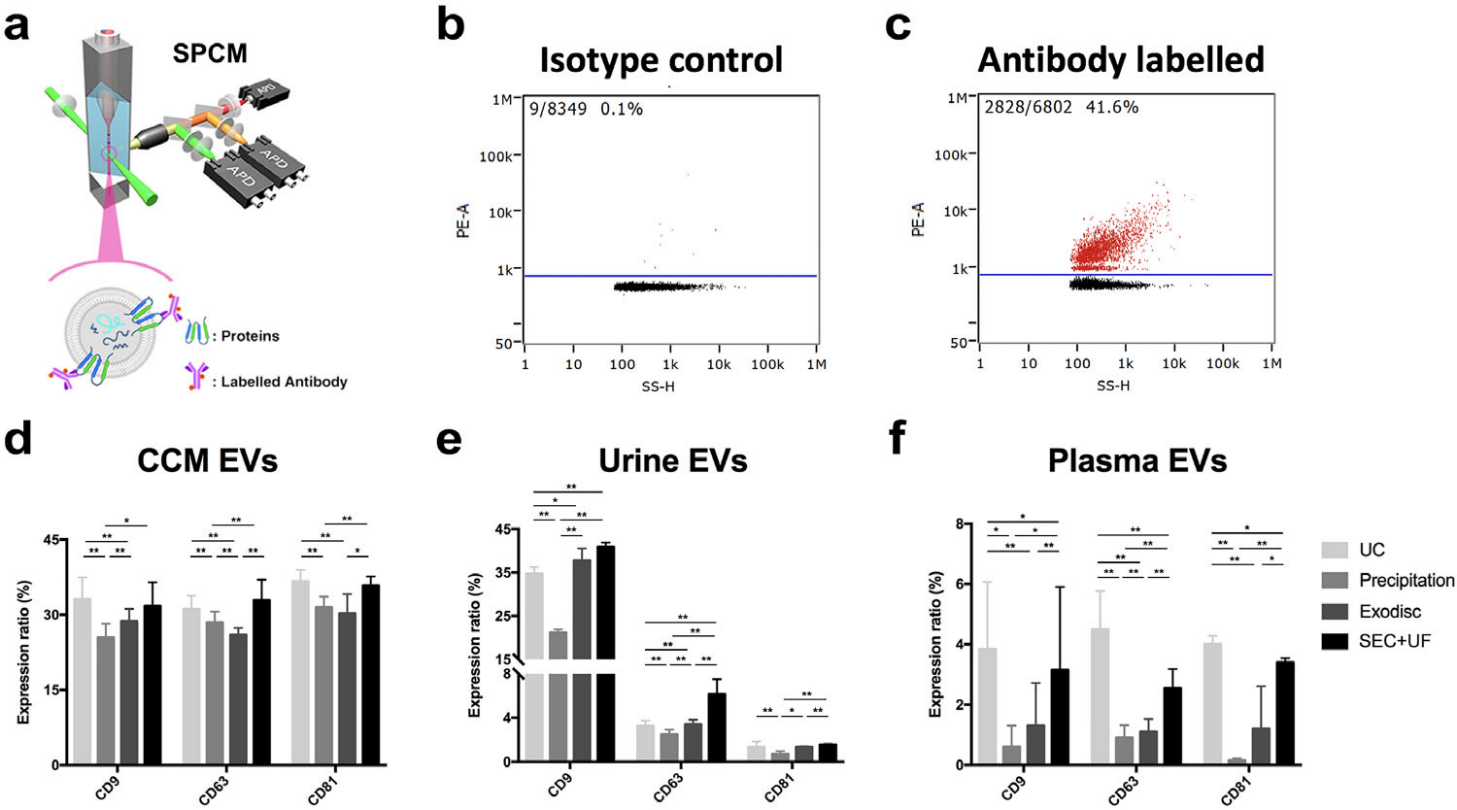
Purity of EV samples assessed by single particle phenotyping using nFCM. (a) Schematic diagram of how expression of CD9, CD63 and CD81 on EVs were measured by immunofluorescent labelling using nFCM. (b) Representative bivariate dot‐plots of PE fluorescence versus SSC for isotype control staining and CD9 antibody staining. The IgG isotype control was stained for each sample to identify background and non‐specific labelling, thus, setting the threshold. nFCM will record the number of total particles that were counted, as well as the number of particles above the threshold (positive particles). (d)‐(f) Measured positive percentages of tetraspanin‐positive particles in EV preparations from CCM, urine and plasma separated by different methods. The error bars represented the standard deviation of three repetitive experiments. *P < 0.05, **P < 0.01, one‐way ANOVA analysis with Tukey multiple comparison test as well as a variance‐covariance model

EVs from biofluids are highly heterogeneous populations. Compared to assessing a large pool of separated EVs as a whole, single particle phenotyping allows precise EV subpopulation analysis. Several tetraspanins (CD9, CD63 and CD81) are widely accepted as EV membrane markers because of their participation in EV biogenesis. We quantified the particle subpopulations that express these tetraspanins by single particle phenotyping. The expression of CD9, CD63 and CD81 were measured by immunofluorescent labelling using nFCM (Figure 7a, Figure S5–S7). For each EV preparation, an IgG isotype control staining was conducted to identify background and non‐specific labelling, thus, setting the gate (threshold) (Figure 7b‐c). In CCM, the percentages of CD9^+^ particles were similar with those of CD63^+^ and CD81^+^ particles (Table S4, Figure 7d). In urine, the percentages of CD9^+^ particles were the highest. The expression rates of CD63 and CD81 were very low (Figure 7e). In plasma samples, the expression rates of these tetraspanins were all very low and these markers were not detectable in samples separated by precipitation or Exodisc (Figure 7f).

**FIGURE 8:**
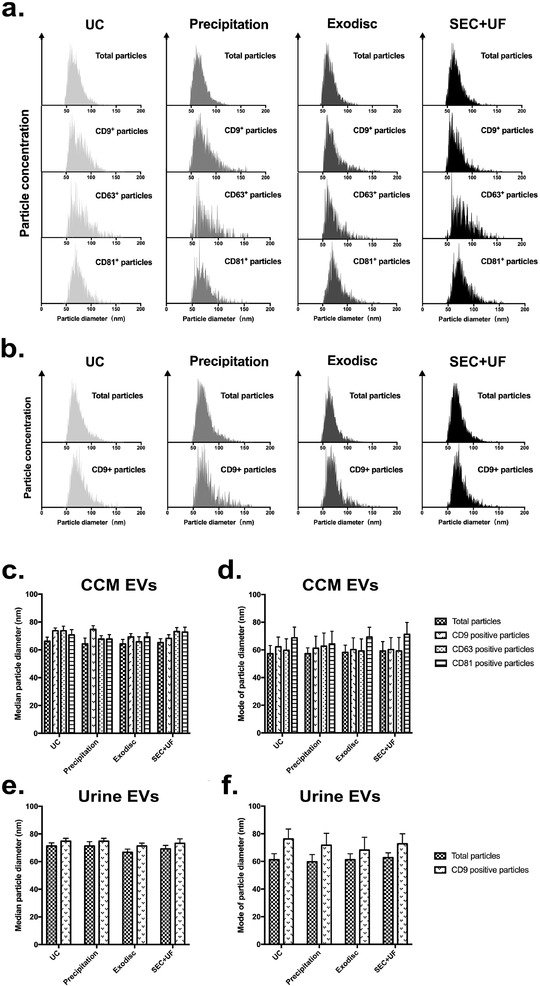
Particle size distributions of the whole EV preparations and tetraspanin‐positive particles from CCM (a) and urine (b) separated by different methods, measured by nFCM. Since at least 1000 tested particles are required for reliable size distribution, only CD9+/CD63+/CD81+ particles in CCM and CD9+ particles in urine were included for analysis. (c) and (d) Median and modal particle sizes of the whole EV preparations and CD9+/CD63+/CD81+ particles from CCM separated by different methods. (e) and (f) Median and modal particle sizes of the whole EV preparations and CD9+ particles from urine separated by different methods. The error bars represented the standard deviation of three repetitive experiments

Total particle size distributions and CD9^+^/CD63^+^/CD81^+^ particle size distributions were assessed by nFCM. Since at least 1000 tested particles are required for reliable size distribution, only CD9^+^/CD63^+^/CD81^+^ particles in CCM and CD9^+^ particles in urine were included for analysis (Figure 8a‐b). Note that these samples were subjected to an additional UC wash following antibody staining, thus, total particle sizes in this analysis were collected from the antibody labelling experiment, which could be different from Figure 2 and 3. In CCM, the median particle diameters were 73.75 ± 2.01 nm for CD9^+^ particles, 73.75 ± 3.25 nm for CD63^+^ particles, 70.75 ± 3.75 nm for CD81^+^ particles and 66.25 ± 2.86 nm for total particles in UC samples; 74.75 ± 2.65 nm for CD9^+^ particles, 67.75 ± 2.46 nm for CD63^+^ particles, 67.75 ± 3.22 nm for CD81^+^ particles and 64.25 ± 4.24 nm for total particles in precipitation samples; 69.25 ± 2.45 nm for CD9^+^ particles, 65.75 ± 3.67 nm for CD63^+^ particles, 69.25 ± 3.10 nm for CD81^+^ particles and 64.25 ± 3.20 nm for total particles in Exodisc samples; 68.25 ± 2.66 nm for CD9^+^ particles, 73.25 ± 2.77 nm for CD63^+^ particles, 72.75 ± 3.54 nm for CD81^+^ particles and 65.25 ± 2.76 nm for total particles in SEC+UF samples (Figure 8c). When comparing modal particle sizes, a better reflection of the size of dominant particles, CD9^+^ and CD63^+^ particles were similar with total particles, while CD81^+^ particles were larger (Figure 8d). In urine, both the median diameters and modal diameters of CD9^+^ particles were larger than those of total particles in all method groups (Figure 8e‐f).

### 3.7 Composition analysis of EV preparations via density gradient ultracentrifugation

**FIGURE 9:**
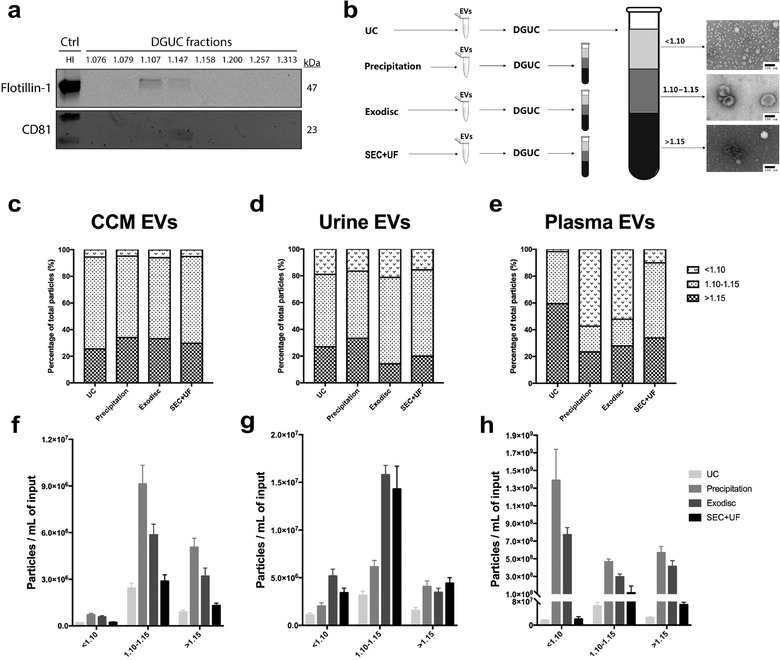
Composition analysis of EV preparations DGUC. (a) Western blots of flotillin‐1 and CD81 for different fractions (*n* = 8) separated from 8 ml of plasma by UC, followed by DGUC fractionation. The same volume of each fraction was loaded regardless of their protein amount. (b) Schematic diagram of the study design and representative TEM images of each density fraction group. Non‐EV particles, like lipids and lipoproteins, were found in low‐density fractions, cup‐shaped EVs were seen in the middle fraction and protein aggregates and other particles were observed in high‐density fractions. Scale bar represented 100 nm. (c)‐(e) Proportions of fraction groups with different densities in EV preparations from CCM, urine and plasma separated by different methods. (f)‐(h) The particle concentration of each fraction group in EV preparations from CCM, urine and plasma separated by different methods. The particle numbers were measured by nFCM. The particle concentrations have been corrected for sample input volumes. The error bars represented the standard deviation of three repetitive experiments

Given the finding that the purity of EV samples varies by different separation methods, it is important to further dissect their composition. DGUC can separate EV preparations to different fractions according to their density. First, we identified the density of EV‐containing fractions as 1.10–1.15 g/ml (Figure 9a). Then we designed an experiment in which EV samples were prepared by different separation methods and then subjected to DGUC. Fractions with a density < 1.10 g/ml, 1.10–1.15 g/ml, > 1.15 g/ml were pooled. Particle numbers in each fraction group were counted by nFCM. TEM images showed that non‐EV particles, like lipids and lipoproteins, were found in low‐density fractions; cup‐shaped EVs were seen in the middle fraction and protein aggregates; and other particles were observed in high‐density fractions (Figure 9b). In CCM, the majority of particles were detected in EV fractions, followed by high‐density fractions. Low‐density fractions contributed the least to the particle population (Table S5, Figure 9c). Compared to CCM, the percentages of low‐density fractions in urinary EV preparations were higher (∼5% vs. ∼15%) (Figure 9d). The density distributions were more diverse in plasma samples. UC and SEC+UF had more particles in EV fractions than precipitation and Exodisc. Both precipitation and Exodisc had a large proportion of low‐density particles (Figure 9e). Figure 9f‐h demonstrated the particle counts of each fraction group in samples separated by different methods. As previously shown, plasma has substantially higher particle yields than other sample types. Precipitation and Exodisc resulted in approximately 100‐fold higher particle yields compared with UC (Figure 1c and f). We found in low‐density fractions, the particle counts of precipitation and Exodisc were 100 times higher than that of UC. However, in EV fractions, they were only 10 times higher (Figure 9h). This experiment gave an insight to the proportion and density of EVs and non‐EV particles in separated “EV samples”. However, since some non‐EV particles (e.g., HDL in plasma) share a similar density with EVs, the EV fractions may contain not just pure EVs. Moreover, some low‐density particles might be lost during the UC wash after DGUC, and therefore the proportion of non‐EV particles in this experiment might be underestimated.

## 4 DISCUSSION

One of the challenges that restricts the evolving field of EV research is the lack of a consensus method for EV separation. This may also explain the diversity of the experimental results in EV research, as co‐separation of soluble proteins and lipoproteins may impede the interpretation of experimental findings (Monguió‐Tortajada, Gálvez‐Montón, Bayes‐Genis, Roura, & Borràs, 2019). Multiple reviews have addressed this concern and have provided qualitative evaluations of separation methods based on published data (Coumans et al., 2017, Monguió‐Tortajada et al., 2019, Xu, Greening, Zhu, Takahashi, & Simpson, 2016). However, quantitative comparisons of EV separation methods in different sample types are still needed. It is also important to further understand the relative proportion of separated “true” EVs versus non‐EVs in sample preparations. In this study, the EV yields and sample purities of three commonly used methods and one microfluidic device were comprehensively evaluated in CCM, urine and plasma.

EV yield, the number of EVs separated from a certain input material, regardless of contamination levels, is a key factor in EV separation method evaluation. This is an important consideration because most of the samples used for EV research have a limited volume, especially the ones from clinical practice (e.g., biofluid from patients). This is also an essential parameter for selecting methods for certain downstream experiments, for example, the wash step after incubation with dye/antibodies in EV labelling assays. Particle yields can hide the true EV yields since each EV separation method may have a certain bias towards subsets of EVs or contamination of non‐EV particles. Spiking experiments using GFP labelled viral particles can elucidate more reliable EV yield of each method because they largely exclude the co‐separation of contaminants in biological samples. In this study, Exodisc have the best EV yield, followed by precipitation, SEC+UF and UC. The recovery rates of UC and precipitation were similar to the published finding, while that of SEC+UF was lower than the reported data (Tian et al., 2020). One of the reasons to explain this could be the additional ultrafiltration step increased the chances for MVP‐GFP to stick to the filter surface. This data also demonstrated that it is important to perform spiking experiments using EV‐mimic particles in a more complexed buffer containing proteins, instead of pure PBS, because it can better reflect the separation efficiency in real biofluids and the proteins may increase the recovery rates by coating the surface. According to the working principle, precipitation may pull down both EVs and other non‐EV particles, thus, it usually has the highest particle yield in biofluids (Helwa et al., 2017, Patel et al., 2019). However, in this study the particle yield of precipitation in urine samples was low. One hypothesis was the protocol used in this study only required 60‐min incubation time for precipitating agent (compared to overnight incubation for CCM), which might not be sufficient. The cost of time should also be considered in terms of efficiency, especially in clinical scenarios, for example, EVs as biomarkers for clinical diagnosis. Both Exodisc and SEC+UF require less than 30 min to perform, while UC takes at least 2–4 h. The time that a precipitation protocol needs may vary in different sample types, but the hands‐on time is generally low.

The purity of EV samples is usually the largest concern because it can fundamentally change the interpretation of experimental results. It is critical to achieve as pure EV samples as possible, especially for certain downstream analysis, like EV proteomics. We found no 100% pure EV preparations due to the overlap of their size and density with many non‐EV particles in biofluids. The sample purity is influenced by both pre‐treatment of the original sample and EV separation steps. Notably, the choice of pre‐treatment protocols can also lead to different results among studies (Merchant, Rood, Deegens, & Klein, 2017, Witwer et al., 2013). Here all the biological samples have gone through the standard pre‐treatment protocol according to MISEV guideline, in which cell debris and other contaminants (e.g., platelets in plasma) were removed (Théry et al., 2018). To increase purity, we discarded the pellets separated by 10,000 × *g* centrifugation that may contain large EVs (e.g., apoptotic bodies, large oncosome, etc.), but also have a higher level of contamination (Collino et al., 2017, Minciacchi et al., 2017).

In this study, multiple experiments were applied to assess the purity of EV samples, including spiking experiment, TEM, particle/protein ratio, WB, single EV phenotyping and composition analysis by DGUC. Each of these has pros and cons and together they give a more comprehensive overview of sample purity. TEM can visualize the EVs and other non‐EV particles but has potential sampling bias since it can only show a small piece of the whole picture. Particle/protein ratio is the first and widely used standard for the purity assessment of EV preparations, but since some large lipoproteins or protein aggregates can also be counted in the particle count, the ratio could overestimate the purity of certain samples (Takov, Yellon, & Davidson, 2019, Webber & Clayton, 2013). Recently, there has been interest in single EV phenotyping by high‐resolution nFCM which can not only demonstrate the heterogeneity of EV subpopulations but can also be adopted to evaluate EV purity by assessing the proportions of tetraspanin‐positive particles (Tian et al., 2018, Tian et al., 2020, Zhu et al., 2014). The advantage of this new assessment is that it detects EV markers on a single‐particle level. The drawback is that non‐EV particles smaller than the lower detection limit cannot be counted by this method and thus, if present, may lead to an overestimation of purity. Given the different antibody affinities and the potential deconjugation of EVs and antibodies during the UC wash after incubation, the proportion of true EVs should be taken as a relative estimation, instead of a read‐out of the exact percentage. Interestingly, both the median and modal particle sizes of tetraspanin‐positive particles were larger than those of total particles in both CCM and urine, indicating that the contaminants were mainly small particles (free proteins or lipids, etc.) or smaller EV may not express sufficient tetraspanins to be detected. Our composition analysis of EV preparations by DGUC allows evaluation of some co‐separating non‐EV particles. It may also allow us, roughly, to estimate the proportion of “true” EV particles. Of course, we recognize that additional analysis of fractions by both imaging and marker detection may be needed and informative. Another important difference from previous studies is that most studies count EV numbers by NTA, of which the minimum detectable EV sizes are 70–100 nm (Van der Pol et al., 2014). Thus, small EVs cannot be accurately measured. However, the minimum detectable EV size is reported to be as small as 40 nm for nFCM, thus capturing a more precise particle count (Tian et al., 2018, Tian et al., 2020).

EVs coexist with a multitude of non‐EV particles in biofluids. The dominant compositions will determine the difficulty of EV separation from a certain biofluid and the main contamination types in EV preparations. The FBS supplement used in most CCM contains significant quantities of exogenous EVs and non‐EV particles. Although in most of EV studies, EV‐depleted FBS was used, it has been demonstrated that it still contributes almost 7×10^9^ countable particles/ml, even though 75% of particles have already been depleted (Lehrich, Liang, Khosravi, Federoff, & Fiandaca, 2018). Typically, protein load is low in human urine (except for patients with various kidney diseases). THP is the main abundant soluble protein in urine that can trap EVs and form aggregates. It's an 85 kDa protein and can be detected in 1.18–1.24 g/ml fractions by DGUC (Dhondt et al., 2020, Fernández‐Llama et al., 2010, Mussack, Wittmann, & Pfaffl, 2019). Plasma is one of the most popular but challenging samples for EV research. It contains a large variety of proteins, lipids, lipoproteins, etc., among which HDL shares a similar density with EVs, while very low‐density lipoproteins (VLDL) and chylomicrons share a similar size (Coumans et al., 2017). This is one reason why it is challenging to purify EVs from plasma regardless of the separation method. In this study, in general, urinary EVs had the highest purities, followed by CCM, plasma, regardless of separation methods. Each separation method may have a bias towards the sample type regarding purity. SEC+UF in general worked well in all sample types; UC can remove the vast majority of contamination, however, the THP/THP‐EV complex can be easily pulled down in urine; both precipitation and filter‐based technology, like Exodisc, may perform better with samples containing fewer non‐EV particles.

UC was the first established technique for EV separation and still the most commonly found in the literature (Gardiner et al., 2016). Although it has been considered as “gold standard” for EV separation, there are several drawbacks of this method. It is labour‐intensive, time‐consuming, operator sensitive and requires specific ultracentrifuge machine, which largely limits its application in clinical setting. Another concern is that centrifugation at such high speeds can negatively affect the intactness of EVs (Baranyai et al., 2015, Linares, Tan, Gounou, Arraud, & Brisson, 2015, Monguió‐Tortajada et al., 2019). We demonstrated UC had the lowest yield among the four assessed methods, which explained the low particle yields in all the sample types. In CCM and plasma, UC had the highest purity among the four methods. However, UC also co‐ separated non‐EV particles. In urine, the purity of UC was suboptimal mainly due to the co‐precipitation of THP which has been confirmed by WB and DGUC fraction analysis (UC had a higher proportion of high‐density contaminants compared to Exodisc and SEC+UF).

Precipitation techniques, the basis for many commercial EV separation kits, are very popular among labs new to the EV field due to its relatively inexpensive and user‐friendly features. Most of these protocols apply the polyethylene glycol (PEG)‐based volume exclusion precipitation. Though the recovery can be high, the purity of precipitated EV samples are usually very low, as it basically precipitates all soluble particles (Konoshenko, Lekchnov, Vlassov, & Laktionov, 2018, Lobb et al., 2015, Rider, Hurwitz, & Meckes, 2016). Rather than an EV separation method, it is more like a concentration tool. The precipitating agent cannot be removed from the final preparation, which may potentially affect the downstream characterization or analysis. In this study, the purity of precipitated EVs was the lowest regardless of sample types and the level of contamination was largely dependent on the intrinsic composition of the input sample. In CCM, the EV markers were weakly detected by WB. However, in plasma, it has been demonstrated that at least 98% of particles were probably non‐EV particles. In urine, both the particle yield and purity were low. Surprisingly, the THP was not detected by WB, but this is consistent with another study (Royo et al., 2016). Theoretically the large amount of THP should be precipitated. The residual precipitating agent may or may not contribute to the absence of THP band. Further study is needed.

Exodisc is the only new technology tested in this study. It is one of the next‐generation microfluidic devices that combines the advantages of both TFF (high efficiency) and microfluidic devices (portable and user‐friendly) (Shao et al., 2018, Woo et al., 2017). In this study, Exodisc showed moderate purity for CCM and high purity for urine, while the purity for plasma EVs was low. Similar to precipitation, the purity of Exodisc separated EVs is dependent on the amount and type of non‐EV particles coexisting with EVs in the input sample, however, the difference is that Exodisc can clean up most of these contaminants if the filter is not overwhelmed (e.g., in urine, the THP was not detected in EV samples). Our data suggested there is a heavy contamination of lipids and/or lipoproteins in EVs separated by Exodisc from plasma, which is consistent with the finding from spiking experiment, that when the input concentration of contaminating protein was high, Exodisc had a very high recovery rate, but co‐separated a large amount of proteins as well. Theoretically these particles are smaller than the pore size and should have been removed. Sunkara and colleagues found by SEM that if there were a huge number of small particles overwhelming the filter, they could form a “bed” and cause clogging (Sunkara et al., 2019). On the other hand, Exodisc may have the best EV yield among all tested methods, and it is very time‐efficient, which allows it to be a good option for separated EV washing in multiple downstream experiments and a candidate for future clinical application, especially for urine.

In recent years, SEC is increasingly being adopted as the second common option for EV separation (Gardiner et al., 2016, Monguió‐Tortajada et al., 2019). The separation by SEC is based on the differential elution profiles of particles with different sizes running through a porous polymer. Small particles (e.g., proteins) enter the pores of the polymer, which delays the flow‐through. It is efficient, inexpensive, user‐friendly and takes a short processing time. Moreover, the gravity flow used in SEC causes minimal damage to EV structure and function (Gámez‐Valero, Monguió‐Tortajada, Carreras‐Planella, Beyer, & Borràs, 2016, Guan et al., 2020). Mol and colleagues investigated the effect of EVs separated by UC or SEC from cardiomyocyte progenitor cells on endothelial cells. They found SEC‐EVs bear higher functionality (Mol, Goumans, Doevendans, Sluijter, & Vader, 2017). The drawbacks of this technique include the limited input volume (∼500 μl in this study) and a dilution effect in EV elution (2 ml in this study). To solve these problems, ultrafiltration is widely used to concentrate the samples before and after SEC (Benedikter et al., 2017, Oeyen et al., 2018). In this study, the purities of SEC+UF separated samples were high in CCM and urine. Urinary THP was not detected by WB. In plasma, though the particle/protein ratio of SEC+UF was higher, the single particle analysis demonstrated a higher EV proportion in UC. Technically, while SEC allows the separation of EV from HDL particles, it cannot fully exclude other lipoproteins like chylomicrons (75–1200 nm) or VLDL (30–80 nm) (Karimi et al., 2018, Sódar et al., 2016). Takov and colleagues found SEC separated EVs contained a large amount of APOB^+^ lipoproteins (Webber & Clayton, 2013). Consistent with these findings, we found a larger proportion of low‐density fractions in SEC+UF samples by TEM images, as well as by DGUC fraction analysis. On the other hand, the particle yield of SEC+UF was almost 20 times higher than that of UC in plasma EV separation, however, in the true EV fractions separated by DGUC, the difference was within 2 folds, implying a large number of countable particles in SEC+UF EV preparations were non‐EV particles.

Besides the technical limitations that have already been addressed in the discussion, there are also some limitations with the study design. All the urine and plasma samples were from healthy donors. The translation of the findings from a variety of disease conditions could be more challenging. In order to make the final results measurable, we used different input sample volumes for DGUC fraction analysis and normalized the results to per ml of input. Given the large differences in particle yields among these methods, this is inevitable, however, may have caused some bias in the calculation. And at last but not least, although the DGUC fractionation gave a reasonable insight of the contamination type, further analysis by proteomics or lipidomics will give a more detailed profile of all these contaminants.

**FIGURE 10:**
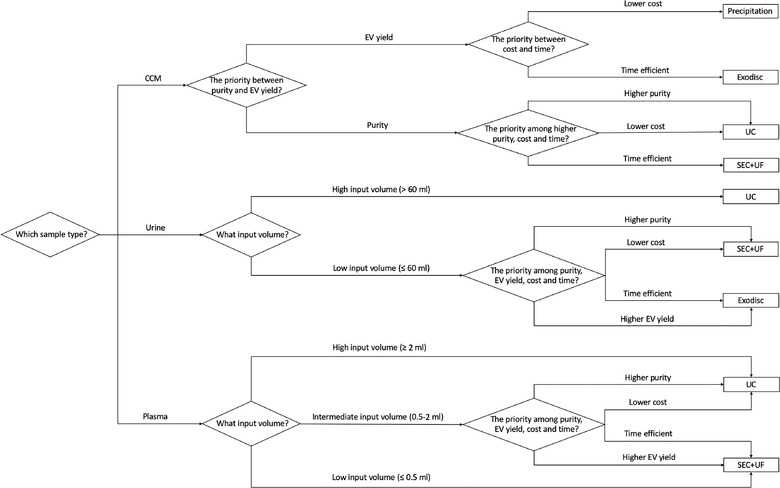
The decision tree on how to select the proper EV separation method based on the input sample. Each diamond box stands for a decision‐making point. For CCM, the first question to consider is the priority between purity and EV yield because the two methods with good sample purity have suboptimal EV yield, while the two have high EV yield can't provide good purity. The sample volume is not a decision‐making point for CCM because for urine and plasma, we usually can't control the volume of the sample, especially in the clinical setting, but for CCM, the volume depends on the need and cell type, which is controllable. For urine and plasma, the volume cutoffs are rough estimations for practice. For example, each Exodisc has six chambers and in each, about 10 ml of urine samples can be processed (depending on the concentration of the urine). The biggest concentrator described in this study can pre‐concentrate up to 70 ml of urine for SEC+UF each time and each SEC column used in this study can load up to 0.5 ml of sample. That doesn't necessarily mean it is impossible to use a certain method with an out‐of‐range input volume. It will just cost more time and materials. The precipitation is not considered as an option for urine, nor are precipitation and Exodisc for plasma, because the sample purity may be too low for any downstream work to have reliable findings

In conclusion, our findings suggest among the four tested methods, Exodisc has the best EV yield though it may co‐separate contaminants when the non‐EV particle levels are high in input materials. Precipitation has the lowest sample purity, regardless of sample type. The purity of the other techniques may vary in different sample types and are largely dependent on their working principles and the intrinsic composition of the input sample. Researchers should wisely choose the proper separation method and find a balance between EV yield and purity according to the sample type, downstream analysis of choice and their working scenarios, for example, clinic or lab (Figure 10 and Figure S8).

## CONFLICTS OF INTEREST

UNIST has filed patents on Exodisc and YKC is named as an inventor, which are licensed to LabSpinner, Inc.

## Supporting information

Supporting Information

Supporting Information

Supporting Information

Supporting Information

Supporting Information

Supporting Information

Supporting Information

Supporting Information

Supporting Information
