# Supplementary figures and images for: Comprehensive evaluation of methods for small extracellular vesicles separation from human plasma, urine and cell culture medium
Source: J Extracell Vesicles. 2021 Jan 15;10(2):e12044. doi: 10.1002/jev2.12044 (PMC7810129; doi:10.1002/jev2.12044)

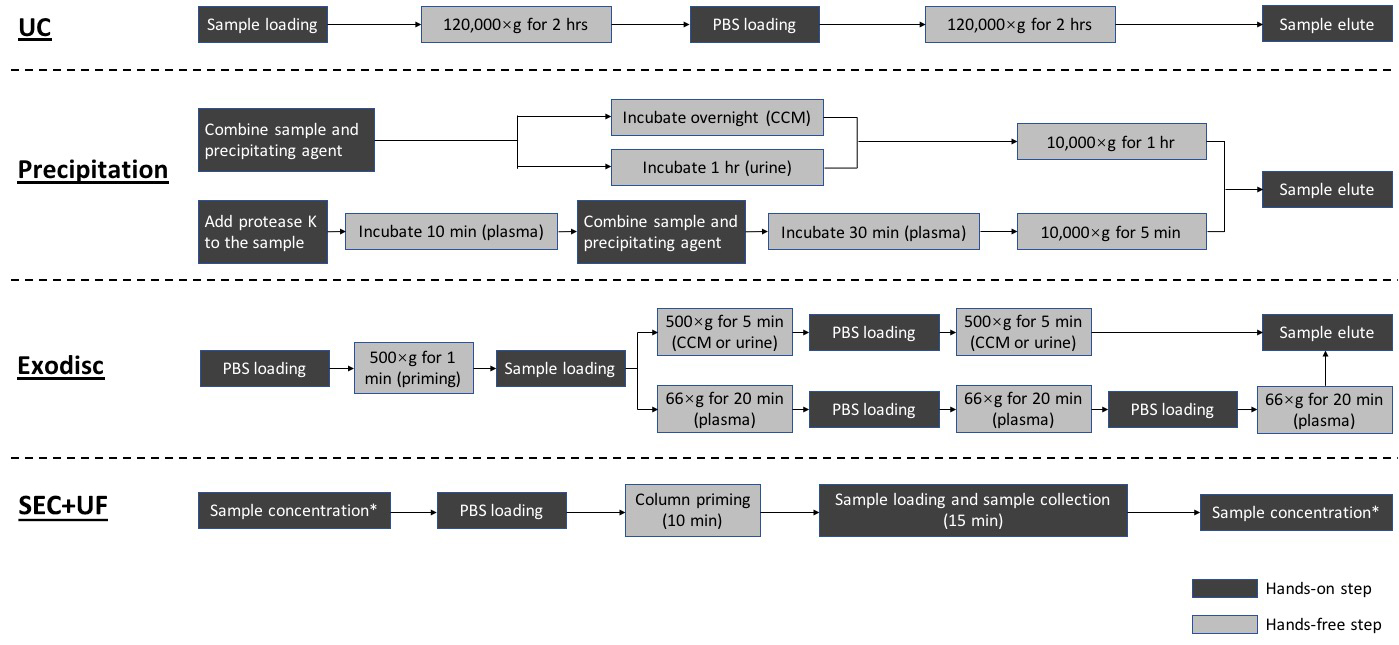

Supplement: Supplementary file 1 — Supporting Information [file JEV2-10-e12044-s001.jpg]

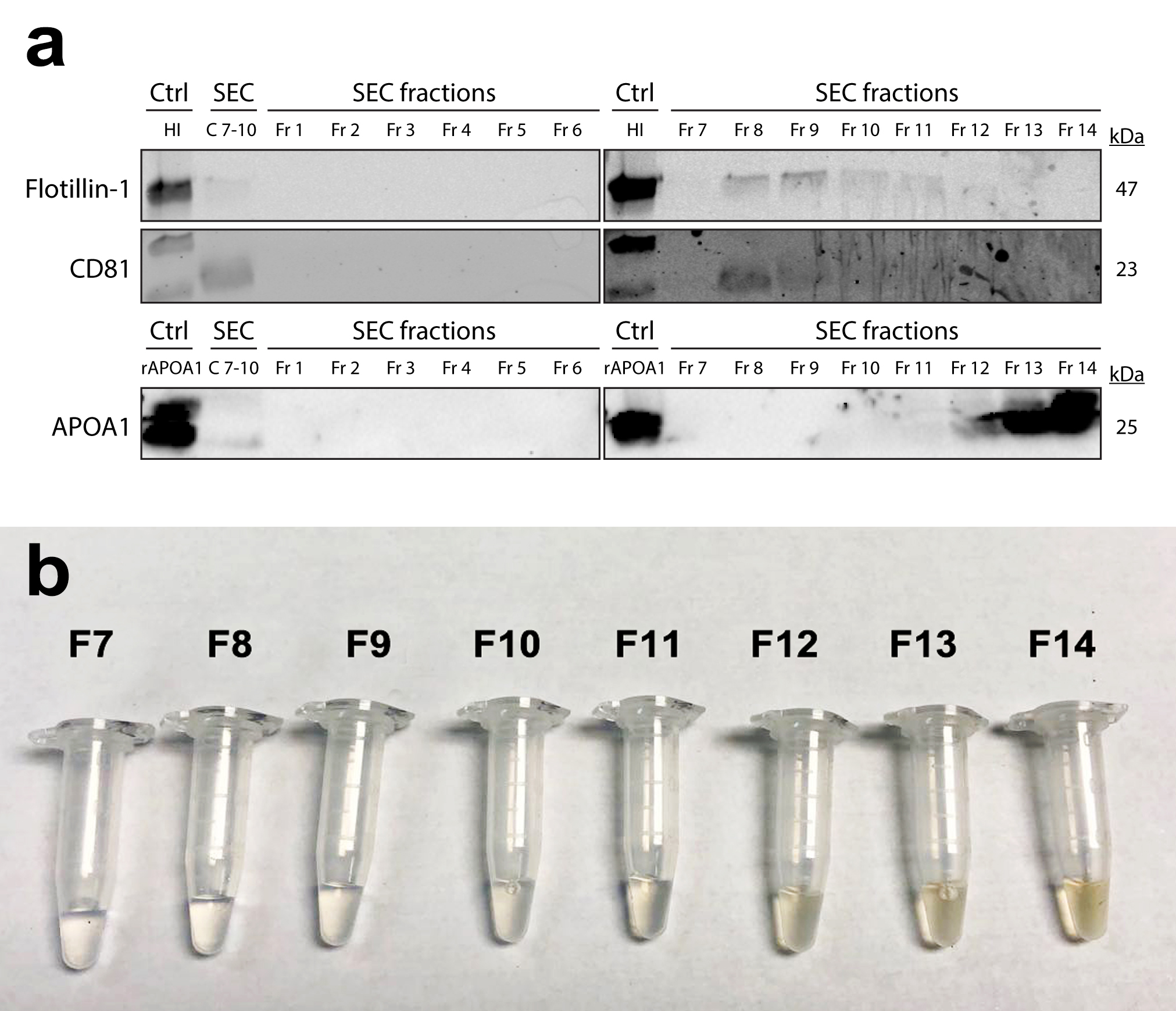

Supplement: Supplementary file 2 — Supporting Information [file JEV2-10-e12044-s002.jpg]

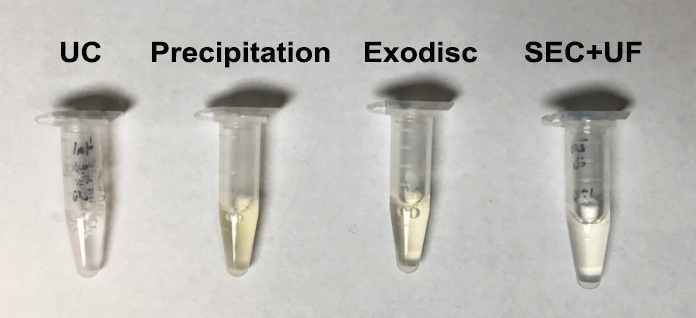

Supplement: Supplementary file 3 — Supporting Information [file JEV2-10-e12044-s003.tif]

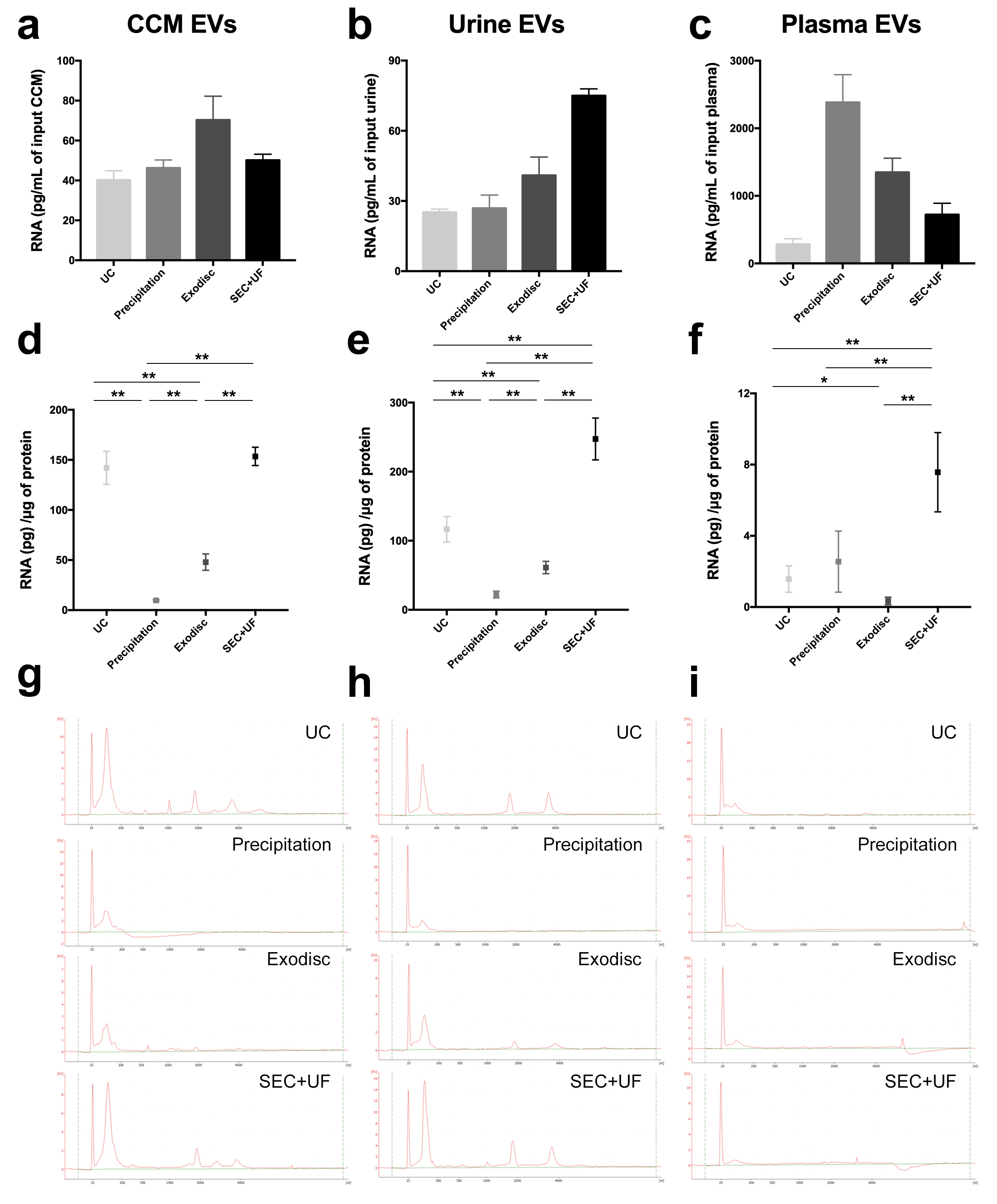

Supplement: Supplementary file 4 — Supporting Information [file JEV2-10-e12044-s004.jpg]

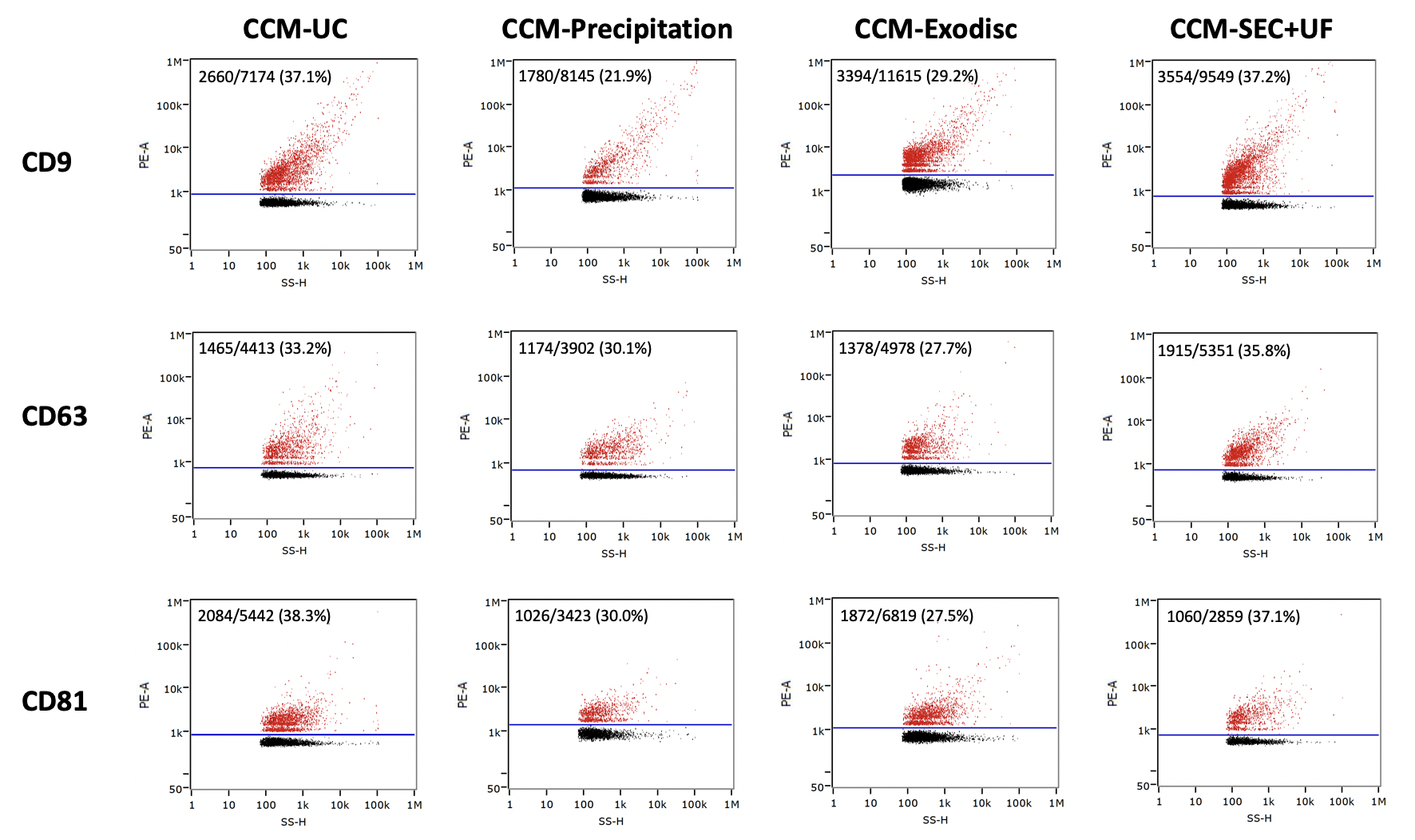

Supplement: Supplementary file 5 — Supporting Information [file JEV2-10-e12044-s005.png]

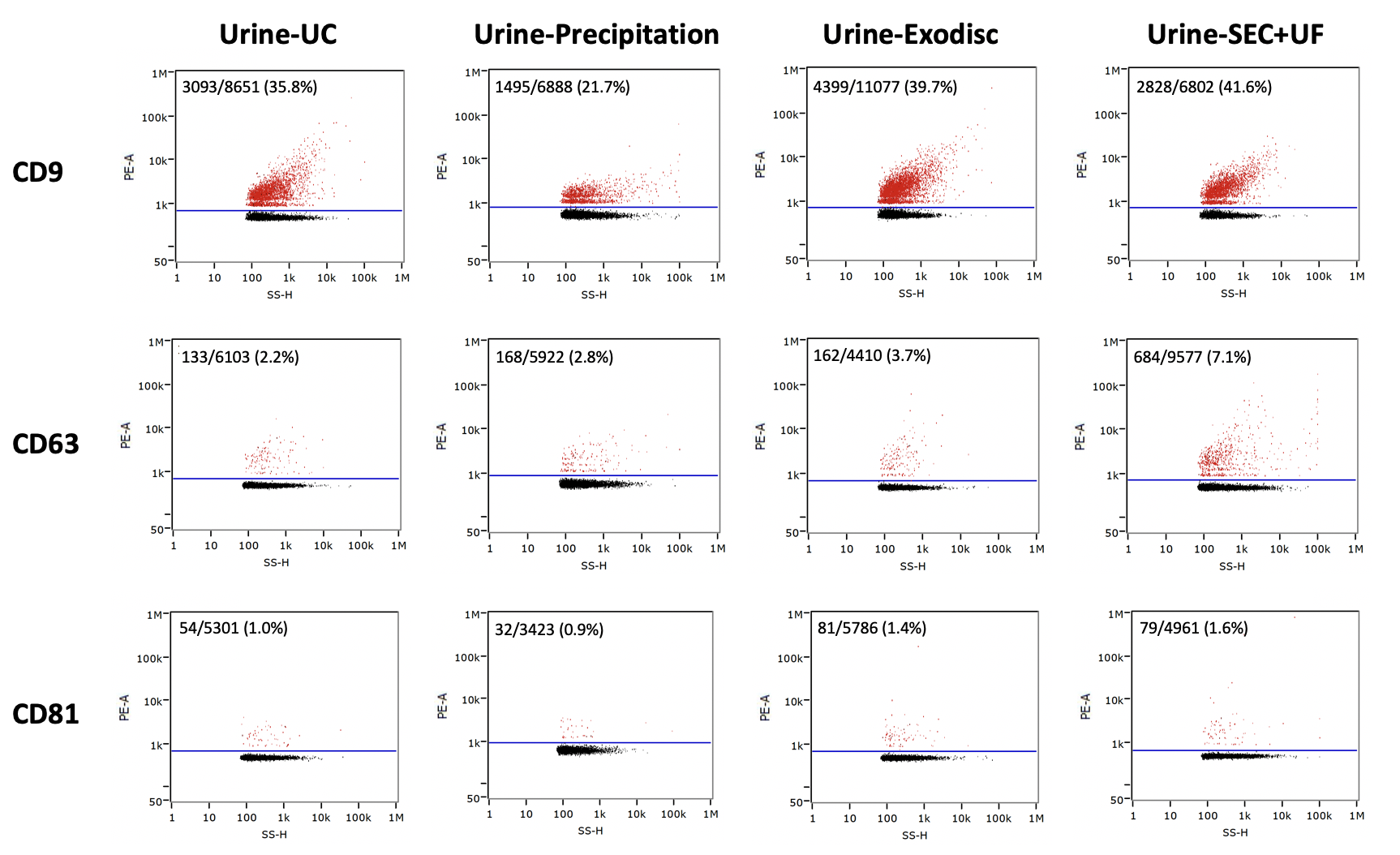

Supplement: Supplementary file 6 — Supporting Information [file JEV2-10-e12044-s006.png]

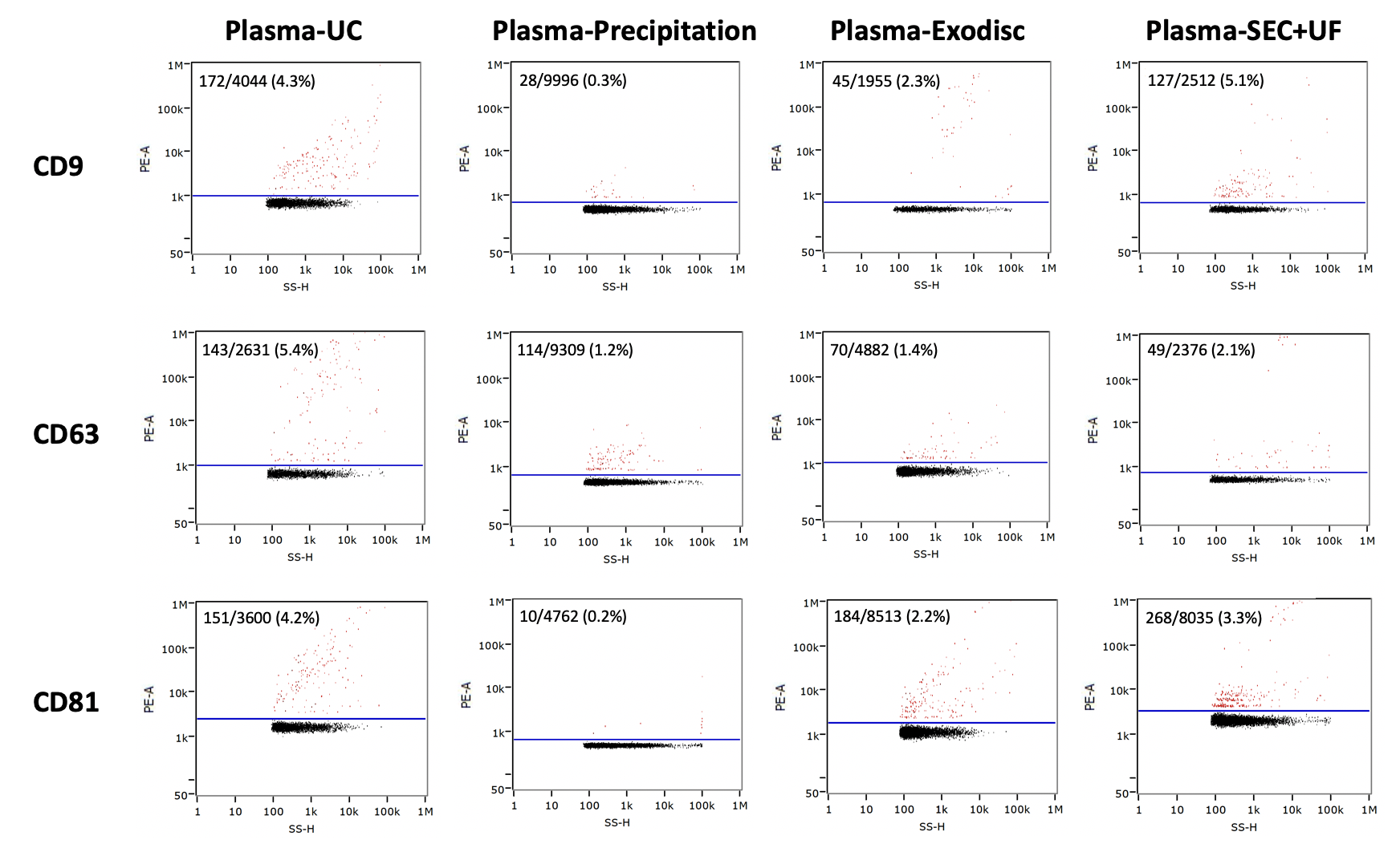

Supplement: Supplementary file 7 — Supporting Information [file JEV2-10-e12044-s007.png]

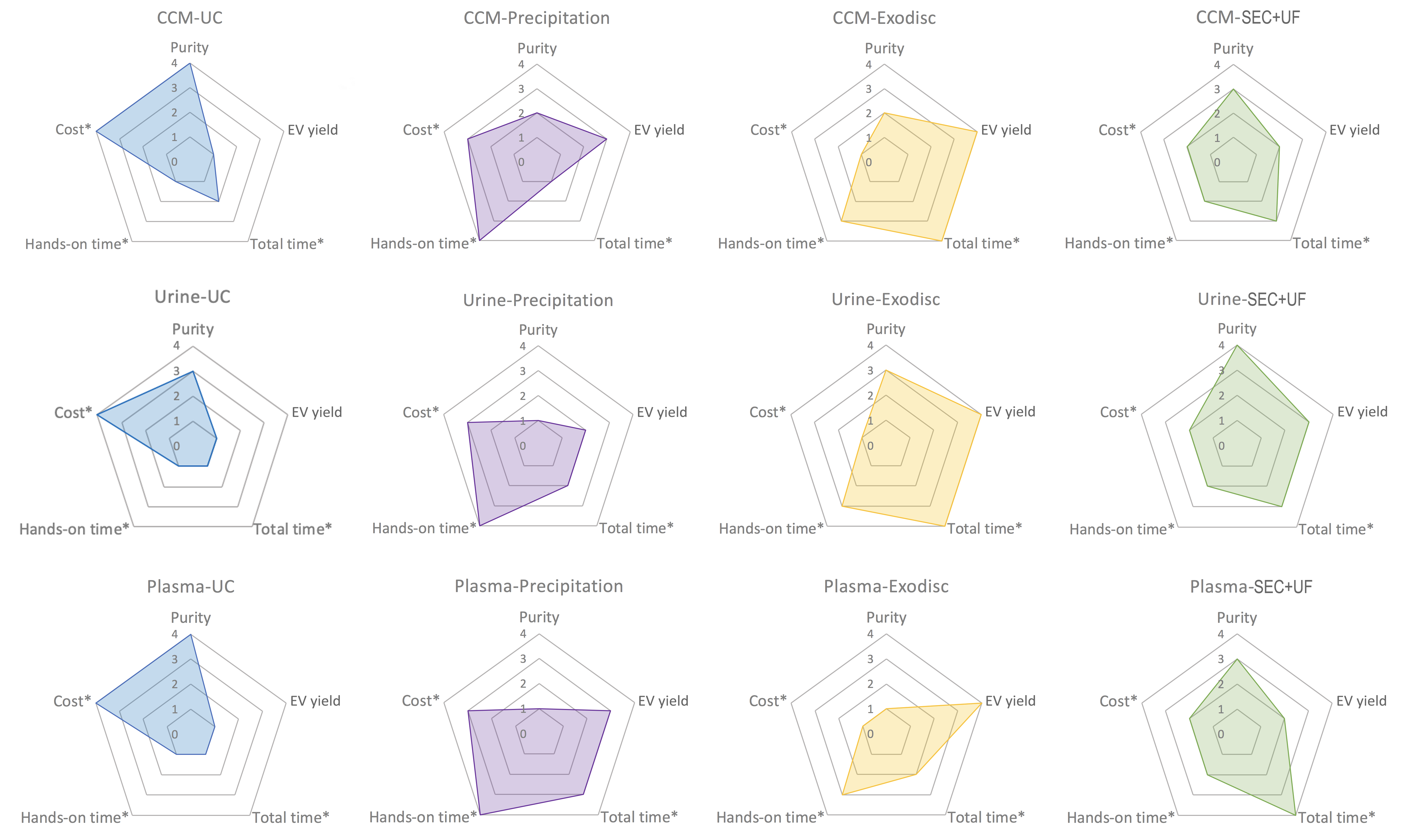

Supplement: Supplementary file 8 — Supporting Information [file JEV2-10-e12044-s008.tif]
