# Supplementary material for: Comprehensive evaluation of methods for small extracellular vesicles separation from human plasma, urine and cell culture medium
Source: J Extracell Vesicles. 2021 Jan 15;10(2):e12044. doi: 10.1002/jev2.12044 (PMC7810129; doi:10.1002/jev2.12044)
Supplement: Supplementary file 9 — Supporting Information [file JEV2-10-e12044-s009.docx]

Supplement table 1 Particle yields of each separation method in different sample types

|  | CCM | | Urine | | Plasma | |
| --- | --- | --- | --- | --- | --- | --- |
|  | NTA | nFCM | NTA | nFCM | NTA | nFCM |
| UC | (9.05 ± 1.53) × 10^7^ | (4.92 ± 0.83) × 10^7^ | (3.48 ± 0.94) × 10^8^ | (4.84 ± 6.27) × 10^7^ | (2.42 ± 2.44) × 10^9^ | (5.20 ± 1.75) × 10^8^ |
| Precipitation | (4.42 ± 1.44) × 10^8^ | (1.65 ± 0.91) × 10^8^ | (6.85 ± 5.09) × 10^8^ | (1.65 ± 2.37) × 10^8^ | (2.70 ± 2.10) × 10^11^ | (1.66 ± 1.04) × 10^10^ |
| Exodisc | (2.19 ± 0.75) × 10^8^ | (9.43 ± 3.74) × 10^7^ | (3.64 ± 1.70) × 10^9^ | (8.86 ± 3.61) × 10^8^ | (2.29 ± 1.31) × 10^11^ | (2.05 ± 0.99) × 10^10^ |
| SEC+UF | (6.73 ± 2.96) × 10^7^ | (4.43 ± 1.94) × 10^7^ | (2.77 ± 0.48) × 10^9^ | (5.72 ± 1.91) × 10^8^ | (1.05 ± 0.50) × 10^11^ | (1.08 ± 0.64) × 10^10^ |

Particle yields have been corrected for sample input volume (particles/mL of input volume).

Supplement table 2 Particle modal sizes (nm) of each separation method in different sample types

|  | CCM | | Urine | | Plasma | |
| --- | --- | --- | --- | --- | --- | --- |
|  | NTA | nFCM | NTA | nFCM | NTA | nFCM |
| UC | 113.5 ± 3.6 | 57.25 ± 2.62 | 83.5 ± 4.2 | 61.25 ± 2.12 | 121.5 ± 3.2 | 58.25 ± 3.66 |
| Precipitation | 110.5 ± 4.1 | 59.75 ± 1.73 | 107.5 ± 2.6 | 59.75 ± 3.22 | 127.5 ± 3.2 | 61.75 ± 2.02 |
| Exodisc | 115.5 ± 3.2 | 57.25 ± 2.17 | 110.5 ± 3.1 | 59.75 ± 3.22 | 129.5 ± 3.5 | 62.25 ± 2.15 |
| SEC+UF | 114.5 ± 3.8 | 59.25 ± 2.02 | 110.5 ± 3.6 | 60.75 ± 1.67 | 128.5 ± 3.1 | 58.25 ± 3.43 |

Supplement table 3 Protein concentrations and particle/protein ratios of each separation method in different sample types

|  | CCM | | Urine | | Plasma | |
| --- | --- | --- | --- | --- | --- | --- |
|  | Protein con. | Par/pro ratio | Protein con. | Par/pro ratio | Protein con. | Par/pro ratio |
| UC | 0.28 ± 0.04 | (1.68 ± 0.49) × 10^8^ | 0.09 ± 0.08 | (5.66 ± 1.60) × 10^8^ | 119.49±42.80 | (4.50 ± 0.54) × 10^6^ |
| Precipitation | 4.75 ± 1.51 | (1.15 ± 0.79) × 10^7^ | 1.21 ± 0.70 | (1.36 ± 0.97) × 10^8^ | 1739.68±459.83 | (7.78 ± 1.00) × 10^6^ |
| Exodisc | 1.47 ± 0.22 | (3.73 ± 1.01) × 10^7^ | 0.67 ± 0.09 | (1.32 ± 0.48) × 10^9^ | 4014.13±637.56 | (2.05 ± 0.59) × 10^6^ |
| SEC+UF | 0.33 ± 0.08 | (1.47 ± 0.38) × 10^8^ | 0.30 ± 0.01 | (1.89 ± 0.73) × 10^9^ | 160.27±14.81 | (4.53 ± 1.64) × 10^7^ |

Protein concentrations have been corrected for sample input volume (µg/mL of input volume).

Supplement table 4 Percentages of CD9^+^/CD63^+^/CD81^+^ particles among total particles for each method in different sample types

|  | CCM | | | Urine | | | Plasma | | |
| --- | --- | --- | --- | --- | --- | --- | --- | --- | --- |
|  | CD9 | CD63 | CD81 | CD9 | CD63 | CD81 | CD9 | CD63 | CD81 |
| UC | (33.13 ± 4.30) % | (31.10 ± 2.69) % | (36.65 ± 2.33) % | (34.70 ± 1.56) % | (3.25 ± 0.49) % | (1.35 ± 0.49) % | (3.83 ± 2.24) % | (4.50 ± 1.27) % | (4.00 ± 0.28) % |
| Precipitation | (25.45 ± 2.76) % | (28.50 ± 2.12) % | (31.50 ± 2.12) % | (21.15 ± 0.78) % | (2.50 ± 0.42) % | (0.70 ± 0.28) % | (0.60 ± 0.71) % | (0.90 ± 0.42) % | (0.15 ± 0.07) % |
| Exodisc | (28.67 ± 2.52) % | (26.00 ± 1.41) % | (30.25 ± 3.89) % | (37.75 ± 2.76) % | (3.40 ± 0.42) % | (1.35 ± 0.07) % | (1.30 ± 1.41) % | (1.10 ± 0.42) % | (1.20 ± 1.41) % |
| SEC+UF | (31.77 ± 4.72) % | (32.90 ± 4.10) % | (35.80 ± 1.84) % | (40.90 ± 0.99) % | (6.15 ± 1.34) % | (1.55 ± 0.07) % | (3.15 ± 2.76) % | (2.55 ± 0.64) % | (3.40 ± 0.14) % |

Supplement table 5 Proportions of fractions groups with different densities for each method in different sample types

|  | CCM | | | Urine | | | Plasma | | |
| --- | --- | --- | --- | --- | --- | --- | --- | --- | --- |
|  | Low-density Fr. | EV Fr. | High density Fr. | Low-density Fr. | EV Fr. | High density Fr. | Low-density Fr. | EV Fr. | High density Fr. |
| UC | 5.5% | 69.0% | 25.5% | 18.9% | 54.1% | 27.0% | 1.7% | 38.9% | 59.4% |
| Precipitation | 4.8% | 61.2% | 34.0% | 16.5% | 50.2%, | 33.3% | 57.3% | 19.2% | 23.5% |
| Exodisc | 6.1% | 60.7% | 33.2% | 21.2% | 64.6% | 14.2% | 52.1% | 19.9% | 28.0% |
| SEC+UF | 5.1% | 65.2% | 29.7% | 15.5% | 64.5% | 20.0% | 10.1% | 56.0% | 33.9% |
